# Altered neural cell junctions and ion-channels leading to disrupted neuron communication in Parkinson’s disease

**DOI:** 10.1038/s41531-022-00324-9

**Published:** 2022-06-01

**Authors:** Saptamita Paul Choudhury, Sarika Bano, Srijon Sen, Kapil Suchal, Saroj Kumar, Fredrik Nikolajeff, Sanjay Kumar Dey, Vaibhav Sharma

**Affiliations:** 1 School of Biotechnology, Kalinga Institute of Industrial Technology, Bhubaneswar, Odisha 751024 India; 2 Dr. B.R. Ambedkar Center for Biomedical Research, University of Delhi, Delhi, 110007 India; 3 Indian Institute of Technology-Kharagpur, Kharagpur, 721302 India; 4 Department of Pharmacy, Panipat Institute of Engineering and Technology, Panipat, India; 5 Deparment of Biophysics, All India Institute of Medical Sciences, New Delhi, 110029 India; 6 Department of Health, Education and Technology, Lulea University of Technology, Lulea, Sweden

**Keywords:** Ion channels in the nervous system, Parkinson's disease

## Abstract

Parkinson’s disease (PD) is a neurological disorder that affects the movement of the human body. It is primarily characterized by reduced dopamine levels in the brain. The causative agent of PD is still unclear but it is generally accepted that α-synuclein has a central role to play. It is also known that gap-junctions and associated connexins are complicated structures that play critical roles in nervous system signaling and associated misfunctioning. Thus, our current article emphasizes how, alongside α-synuclein, ion-channels, gap-junctions, and related connexins, all play vital roles in influencing multiple metabolic activities of the brain during PD. It also highlights that ion-channel and gap-junction disruptions, which are primarily mediated by their structural-functional changes and alterations, have a role in PD. Furthermore, we discussed available drugs and advanced therapeutic interventions that target Parkinson’s pathogenesis. In conclusion, it warrants creating better treatments for PD patients. Although, dopaminergic replenishment therapy is useful in treating neurological problems, such therapies are, however, unable to control the degeneration that underpins the disease, thereby declining their overall efficacy. This creates an additional challenge and an untapped scope for neurologists to adopt treatments for PD by targeting the ion-channels and gap-junctions, which is well-reviewed in the present article.

## Introduction

Parkinson’s disease (PD) is a progressing neurological ailment that causes mortality and impacts 1–3% of the worldwide community around the age of 60 years^1^. There are two main types of PD: hereditary, which is biologically acquired either in an autosomal dominant or recessive way, and sporadic (idiopathic), which is thought to emerge through genomic and environmental interactions^2^. Genetic PD accounts for 10–15% of overall occurrences, while all others are categorized as sporadic^3^. There are seven known causative alleles for familial PD: α-synuclein (SNCA), glucocerebrosidase (GBA), leucine-rich repeat kinase 2 (LRRK2), vacuolar protein sorting-associated protein 35 (VPS35), parkin RBR E3 ubiquitin-protein ligase (PARK2), phosphatase, and tensing homolog-induced kinase 1 (PARK7)^4,5^. PD is pathologically characterized by the loss of dopaminergic neurons in the substantia nigra pars compacta (SNpc) accompanied by Lewy bodies, i.e. inclusion bodies rich in α-synuclein^6^. In addition to other mechanisms, α-synuclein also forms an amyloidogenic, helix structure that interacts with membrane lipids of the brain and this interaction can be exploited further for the treatment of PD. The pathological hallmarks of PD are gait freezing, tremor, rigidity, bradykinesia, and postural instability, out of which a known classical triad is tremor, bradykinesia/akinesia, and rigidity/postural instability^7^. Studies on the central nervous system (CNS) have shown that a hundred billion nerve cells^8^ are interconnected and communicate via synapses and thus mediate complicated signaling networks among themselves^9^. Within this complex neural network, gap-junction-based synaptic vesicles remain scattered, thereby playing an important function in the developmental processes of the CNS. Gap-junctions consist of connexins (Cxs) in vertebrates and innexins in invertebrate organisms. Connexins are a broad group of membrane-spanning peptides which facilitate cell-cell interaction as well as the exchange of ions and tiny signaling chemicals^9^. These gap-junctions act as a connection between the nerve cells and glia, namely astrocytes, microglia, and oligodendrocytes. Therefore, many structural or functional alterations in these junctions may lead to PD pathogenesis^10^.

The blood-brain barrier (BBB) remains intact during CNS diseases like PD^11^. The 1-methyl-4-phenyl-1,2,3,6-tetrahydropyridine (MPTP)-model of PD exhibited an increase of a specific Cx expression in the striatum, although the coupling of astrocytes was not increased^12,13^. The alteration of gap-junctions in the brains of PD patients has not yet been reported. Therefore, the pathological role of gap-junctions in PD is still ambiguous. Although most of the mechanisms of tremors and dyskinesias which are commonly observed in PD patients are still obscure, the inferior olive has been focused on as the pathological generator of tremors^14,15^. Neurons of the inferior olive are electrically coupled through gap-junctions, which play a role in creating oscillatory activity. Some studies reported that the Gap-Junction Intercellular Communication (GJIC) of inferior olive neurons is responsible for tremors. However, no difference in the severity of tremor induced by harmaline was observed between Cx36 knockout (KO) mice and wild-type mice^13,14,16^. Neurons, astrocytes, oligodendrocytes, and microglial cells express a diverse set of Cxs which are required during intercellular interaction and the transport of small molecules, ions, as well as neuronal and glial transmitters^17^. They not only play an important function in the CNS but also play a pathological role in many neurodegenerative diseases like PD, Alzheimer’s Disease (AD), and Huntington’s Disease (HD). Many Cxs are substantially upregulated in PD, while a few are negatively regulated. In PD, increased production of some Cxs in the cerebral endothelial cells has been linked to capillary leaking and improved endothelial cell interaction. Similarly, most ion-channels control their firing action potential and the pivot-like activity of dopamine, released from the axonal terminus of subcortical basal ganglia, functions like a motor^18^. For example, positively charged potassium is responsible for maintaining balance and equilibrium within the cell thereby maintaining its volume and at the same time maintaining the potential of the cellular membrane^18^. All of these play a major role in the spreading of PD. Therefore, the current article reviews a diverse array of gap-junctions and ion-channels responsible for PD and their mechanistic overview to address the need for a better understanding of the cause of PD pathophysiology.

Among environmental factors, several occupations such as agriculture, working with pesticides, and heavy metals have higher risks of PD. Studies conducted on highly populated urban areas have also found significant correlations between industrial airborne heavy metal pollution and ambient air pollution from traffic, and an augmented chance of PD onset^10^. Other investigations have associated rural residency as a causative factor for idiopathic PD^3^. Individual genomes are all distinct, and people exposed to the same environmental factor are sometimes impacted differentially, generating a wide range of illnesses including PD^3^. Both the combination impact of hereditary as well as socio-ecological variables might affect the development of individual illness by compositionally changing DNA^3^. Caffeine, for instance, is an adenosine A2A (ADORA2A gene) receptor antagonist that promotes dopaminergic neurotransmitter release^19^, and ADORA2A polymorphism has been reported to lessen the incidence of PD^19^. Pesticide compounds and toxic heavy elements, on the other hand, have a detrimental impact and exacerbate PD by producing gene variants connected to genetic PD (e.g. PARK1, LRRK2 (Leucine-rich repeat kinase 2), PINK1 (PTEN-induced kinase 1)), which culminate in PD-associated processes such as mitochondrial malfunction, oxidative stress, and peptide disintegration impairments^20^. Nevertheless, genetic or environmental factors: both target the gap-junctions and ion-channels of the CNS to produce PD pathogenesis which has thus been reviewed in detail in this article.

Many advanced drugs namely, carbenoxolone, octanol, tonaberasat, and technologies like electroencephalogram, and electrocardiogram are being used to detect and decrease the progression of PD, albeit with incomplete cure or understating of the disease. Thus, we have discussed such drugs and their mechanisms of action in detail in this review to identify and apply newer strategies to treat PD. Overall, an emphasis on the roles of gap-junction and ion-channels in PD is given in this review thereby treating them as useful drug targets to combat this neural disorder. Therefore, the communication between neural gap-junctions and ion-channels, leading to the spread of PD, is discussed in detail and its importance for better physiological and clinical analyses are also explained.

## Parkinson’s disease overview and Braak staging of Lewy pathology

PD, often known as Parkinson’s syndrome, is a protracted neurodegenerative illness that involves damage to the nervous system resulting in motor function impairment. The very first comprehensive explanation of Parkinsonism was presented almost two hundred years ago yet the concept about the progression of the disease keeps changing. Increasing depletion of a dopaminergic neuron inside the SNpc is a critical pathogenic characteristic of PD. Medical correlation analysis revealed that intermediate to extensive dopaminergic neuronal depletion within the SNpc region seems to be the starting point of motor characteristics, particularly bradykinesia and stiffness, in advanced PD^7^. α-synuclein protein remains in a misfolded form in PD. These proteins turn out to be hydrophobic and form subcellular clumps within the cellular body known as Lewy bodies (LBs) and further develop as Lewy neurites of neurons^21^. It has been postulated that Lewy pathology proceeds stereotypically within PD.

**Fig. 1:**
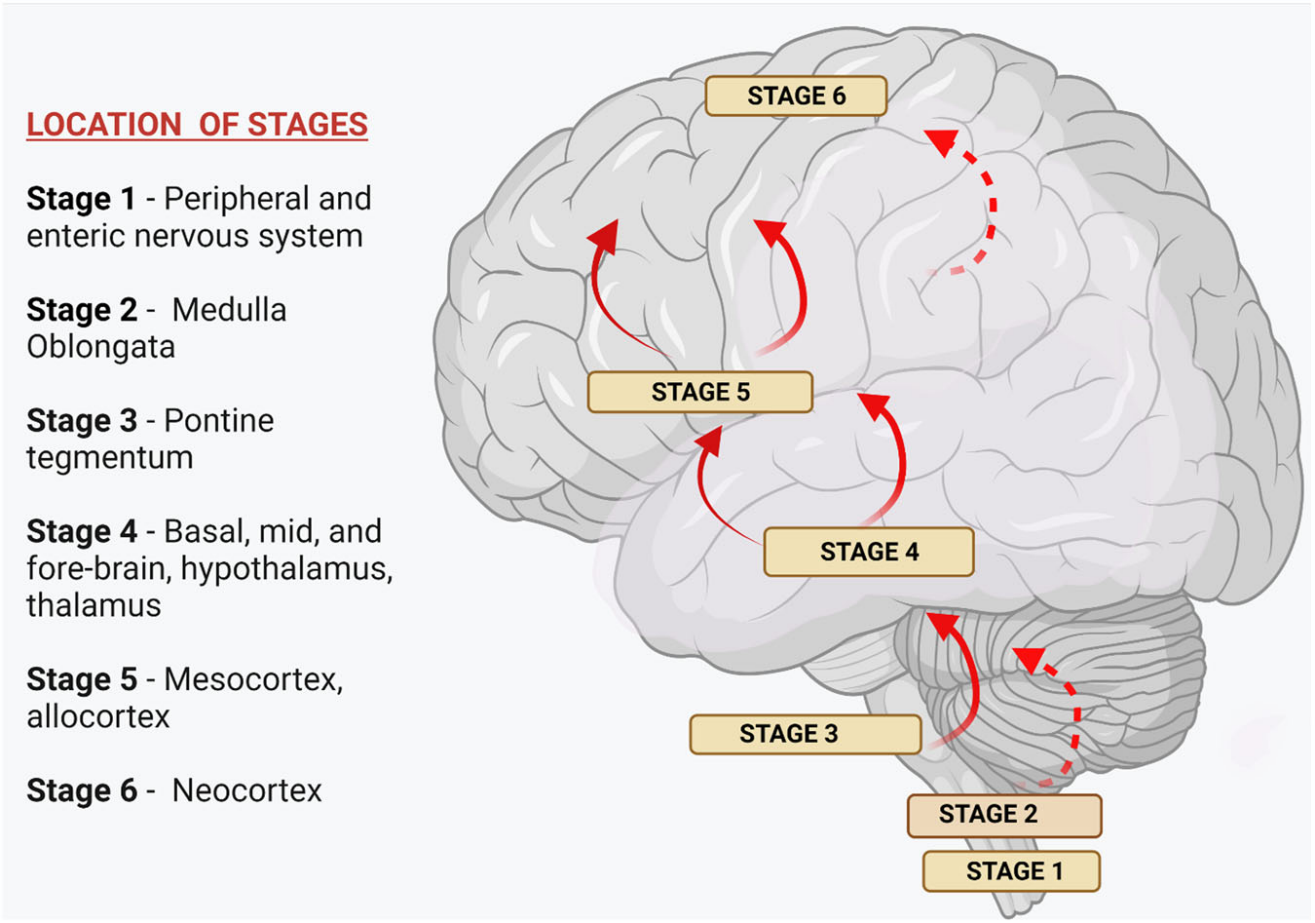
Braak-staging of Lewy pathology in PD. Braak et al., 2003 suggested a six staged course of Parkinson’s illness^203^. As per this concept, α-synuclein accumulates particularly in cerebral areas as well as in neuronal areas, resulting in Lewy pathogenesis throughout a stereo typical, sequential behavior which steps up caudo-rostrally. In its first stage, Lewy pathogenesis starts through the lower midbrain (along with the dorsal motor nucleus of the vagus nerve in the medulla), then to the coeruleus-subcoeruleus complex, raphe nuclei, giganto cellular reticular nucleus in the anteromedial temporal mesocortex, cingulate cortex, and later to the neocortical structures. The illness is thought to initiate in the peripheral and spread to the CNS. With the extent of this Braak stage, the extent of infections in the sensitive areas worsens, and the illness progresses^203^.

Braak and his coworkers have suggested that there are six stages of the progression of PD and it is known as Braak staging of Lewy pathology in PD or Braak hypothesis (Fig. 1). It follows the route from the peripheral nervous system (PNS) to the CNS^3,22^. Phases 1 and 2 might correlate to the start of premotor indications, while phase 3 indicates the presence of motor symptoms owing to nigrostriatal dopamine insufficiency, and phases 4–6 to the presence of non-motor symptoms of severe illness^7^ (Fig. 1).

Olfaction abnormalities, cognition impairments, neuropsychiatric disorders, disturbed sleep, autonomic dysfunction, ache, and exhaustion are examples of non-motor symptoms. Such indications are typical within the initial stages of PD and are linked to low standards of living^23^. The premotor or prodromal stage of the illness is characterized by poor olfactory, bowel problems, depressive symptoms, extreme drowsiness during the day, and quick ocular mobility sleeping behavioral disorders^7^. PD patients have numerous motor impairments, such as gait disruption, poor writing griping strength, even difficulty with speaking, and also a combination of cardinal abnormalities, also known as a classical triad, is formed by tremor, bradykinesia/akinesia, and rigidity/postural instability^24^.

## Etiology of PD: environmental and genetic factors

A majority of PD cases are thought to have a complex etiology resulting from the interaction of environment and genetic variables. Pesticide contact, agricultural labor, or countryside housing have now been linked to an elevated risk of PD which has been proved in various research analyses across the globe. Agrochemicals linked to Parkinsonism, such as paraquat, rotenone, 2,4-D, and many dithiocarbamates and organochlorines, produce exploratory PD in investigational research lending support to the concept that all these correlations represent causative effects^25^. Employment as welders had indeed been related to an elevated danger of PD, presumably as the effect of manganese within welding gases^26^. Nevertheless, research on this link between welders and PD is conflicting. Additional metal ions, including irons and leads, have been linked to an increased probability of PD in exploratory in vitro and in vivo investigations^25,26^. Head trauma could result in a breach of blood-brain barriers, long-term head inflammatory processes, disturbance of mitochondrial function, and an upsurge in glutamine release and α-synuclein buildup within the cortex, all of which would lead to an elevated probability of Parkinsonism^25^. Interestingly, many of our food habits and lifestyle also induces a level of PD. People with high consumption of dairy products, alcohol, and smoking have been proved to show a linkage in the elevated level of PD during many research analyses. There are several potential hazard variables for Parkinson’s in which data is either lacking or conflicting. Initial stage determinants include the time of conception, body mass index during birth, maternal age, and numerous diseases including measles, CNS infections, hepatitis C, and *Helicobacter pylori* are among them. Influenza has already been linked to a higher possibility of PD^27^. However, mechanisms of action of these environmental factors including toxic chemicals on PD are mostly unknown so far.

Many genetic factors and their mutations have been associated with the onset of PD. The earliest gene to be identified and discovered was the SNCA gene which encodes the protein α-synuclein^28^. The leading mutation which is involved in the progression of PD is a missense mutation, which leads to amino acid substitution and multiplication^29^. This amino acid substitution helps in α-synuclein accumulation^29^.

## Pathophysiology of Parkinson’s disease

**Fig. 2:**
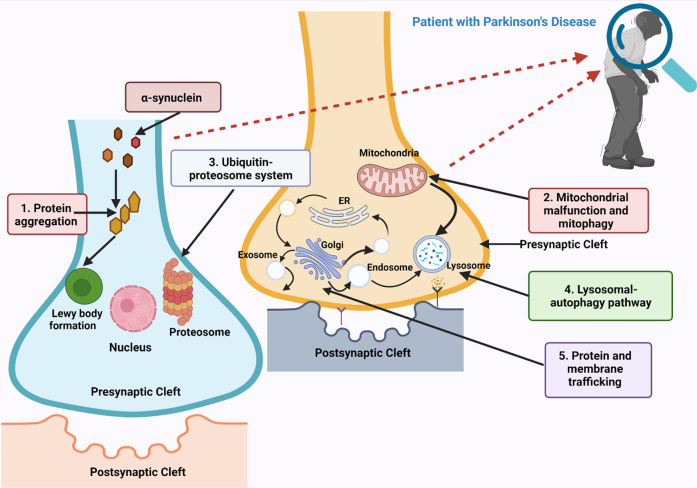
Different molecular pathways that are involved in the pathogenesis of PD. Several factors are implicated in the preferential degradation of substantia nigra neurons in the brain during Parkinsonism, involving cytotoxic **peptide buildup and aggregation** (**1**), **mitochondrial malfunction** (**2**), and oxidative stress^204^. The buildup of α-synuclein is an important phase in pathogenesis which is a component of the Lewy Body. Defects in the **Ubiquitin-Proteasome System** (**3**), and **Lysosomal autophagy pathway** (**4**), which usually operates as a form of protein degradation system, cause changes in protein regulation, which may encourage the extremely toxic buildup of peptides that are harmful to neurotransmission^205^. Neural cell apoptosis in Parkinsonism is also caused by mitochondrial malfunction, which causes an increase in oxidative stress and a decrease in ATP synthesis. Neuronal malfunction and the buildup of misfolded α-synuclein originate from impairments within **protein and membrane trafficking mechanism** (**5**)^206^. Similarly, alterations in intracellular sorting and disintegration have a significant impact on the neuronal trafficking of protein aggregates, enabling the cell-to-cell dissemination of dangerous α-synuclein molecules leading to increased pathological alternations in this disease. ER: Endoplasmic Reticulum.

**Fig. 3:**
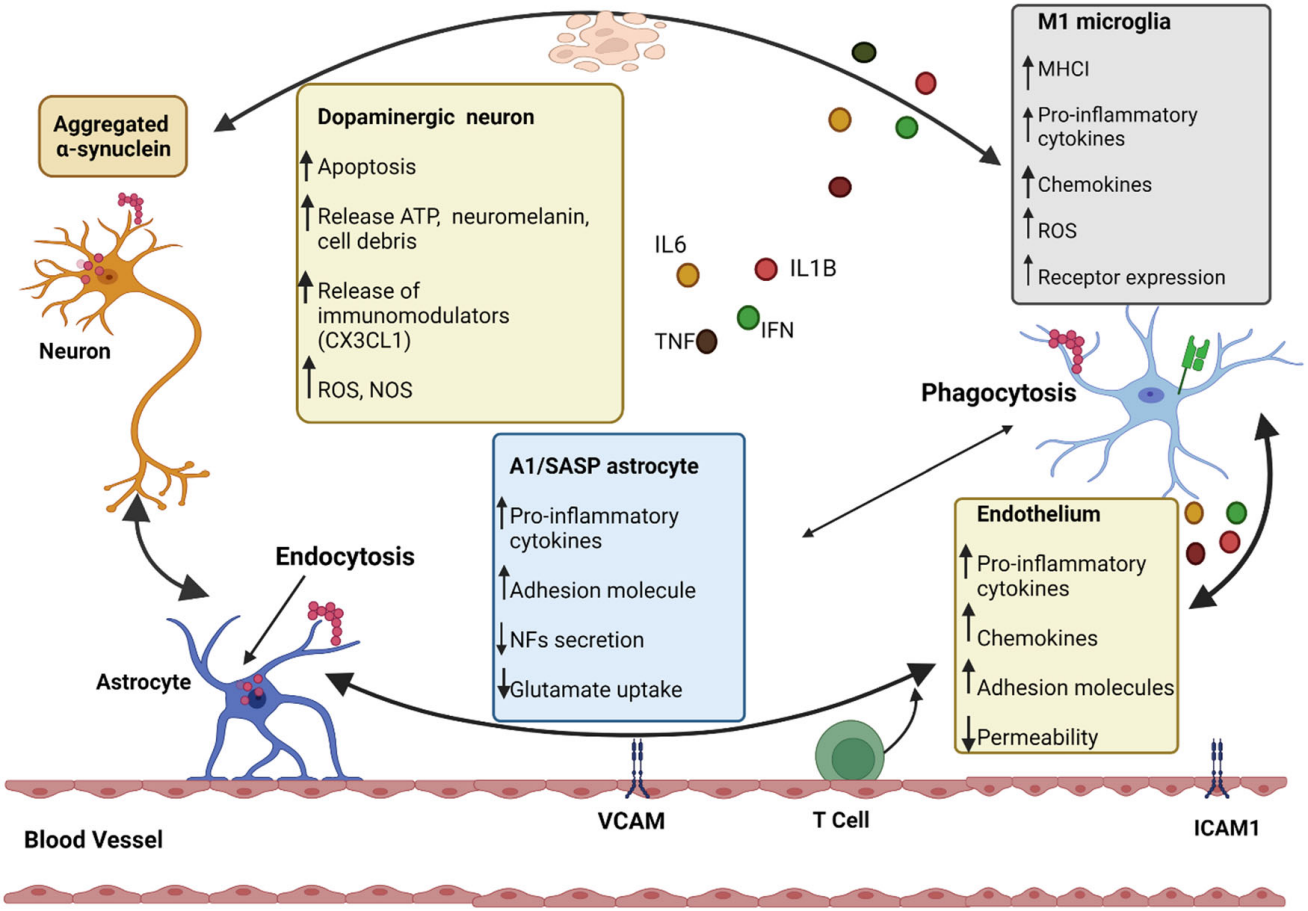
Mechanism of neuroinflammation^207^ in PD. Interaction among various cell groups inside the cortex causes inflammation is depicted here. Neurons, astrocytes, microglia, and endothelial cells are vulnerable to α-synuclein aggregation (i.e., through phagocytic cells, endocytic pathway, Toll-like receptor (TLR) activation, etc.), which could indeed hinder their homeostatic operations (diminished secretion of neurotrophic factors (NFs), deficient glutamate uptake, etc.) as well as production of proinflammatory cytokines and chemokines (MHCI in microglial cells, adhesion molecules in the endothelium, etc.). Peripheral immune cells (such as CD4^+^ T cells) are also drawn into the cerebral tissue. The presence of these immunoregulatory factors, as well as the lack of effective relieving processes, adds to the inflammation milieu. VCAM: Vascular Cell Adhesion Protein, ICAM1: Intercellular Adhesion Molecule 1, NOS: Nitric oxide species, ROS: Reactive Oxygen Species, SASP: Senescence-associated secretory phenotype.

**Fig. 4:**
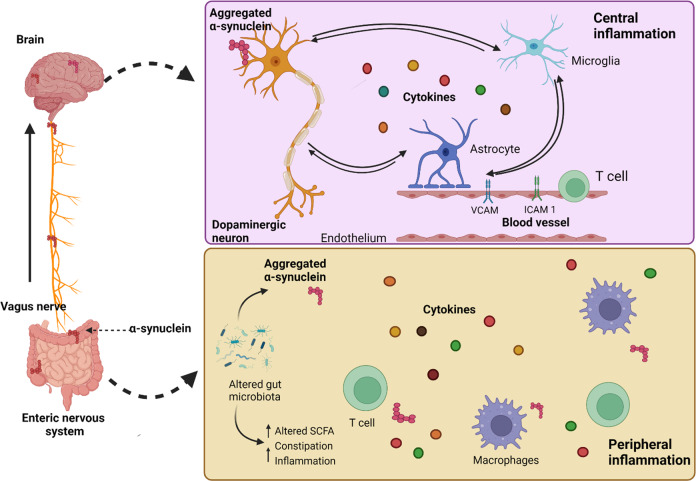
Gut-brain microbiome interaction^207^. Changes inside the intestinal microbiome might encourage α-synuclein accumulation as well as cause an inflammatory reaction inside the periphery. This may include elevated cytokine production and activation of T cells, according to the “gut-brain axis” concept in PD. Aggregation of α-synuclein is thought to propagate in a prion-like way initially from the peripheral to the central nervous system via the vagus nerve. When it enters the brain, proteinopathy, in combination with many invoking variables like mitochondrial impairment, ROS, etc., would then endure core inflammatory response inside a vicious spiral among dropping dead dopamine neuronal cells, glia, and activated endothelium. This will in turn get exacerbated by intruding peripheral immune cells. SCFA: Short-chain fatty acids, VCAM: Vascular Cell Adhesion Molecule, ICAM1: Intercellular Adhesion Molecule 1.

Irrespective of the underlying pathologies (ecological, genomic, or some additional risk variables) of PD, numerous important biochemical processes and characteristics have been identified: (1) in vivo: in adult postmortem samples, human cerebral tissue and organs, and in animals models; and (2) ex vivo: in adult cell lines and cultures. Based on such findings, the pathophysiology of PD may arise from any one or combination of five main possible pathways, namely: (1) α-synuclein protein aggregation mediated pathway, (2) neuroinflammation mediated pathway, (3) neuronal abnormalities, (4) mitochondrial abnormality and mitophagy, and (5) gut-brain microbiome interaction **(**Figs. 2–4**)**. Each of these pathways has specific substages as well, as discussed briefly in this section. However, the role of gap-junctions and ion-channels in PD pathophysiology is less understood or less explained elsewhere which forms the rationale for writing the current article.

### Mechanism 1: α-synuclein protein aggregation mediated pathway

The pathogenesis of PD involves various steps like an agglomeration of proteins, intercellular proteins as well as membrane translocation, ubiquitination, proteasomal pathway, and autophagosome system **(**Fig. 2)^7^. A significant discovery in recent times has shown that PD pathogenesis is mostly caused by α-synuclein via the formation of LBs as explained above. α-synuclein is currently believed to generate a multitude of distinct aggregation forms, comprising of tiny dot-like or narrow ribbon-like structures, aqueous oligomers consisting of 2–100 α-synuclein monomers^30^. All these alternative forms of α-synuclein protein monomers turn out to be toxic to the neurons and result in neurodegeneration in PD. In some PD patients, an inclusion body composed of different proteins is also observed frequently. β-amyloid plaques and tau-containing neurofibrillary tangles which are mainly found in patients with AD are also found in a comparable amount of PD patients.

Due to biological interconnections, α-synuclein is naturally expanded and takes on a tertiary configuration. The protein’s aberrant agglomeration has been known to be harmful to dopaminergic neurons, resulting in neurodegeneration related to PD. α-synuclein structural alterations and accumulation can be influenced by oxidative damage, PD gene mutation**s**, and upregulation^31^. In PD, α-synuclein deposition in astrocytes leads to microglia owing to the production of pro-inflammatory cytokines such as tumor necrosis factor-γ (TNF-γ), interleukin-1 (IL-1), and interferon-gamma (IFN-γ)^30^. In that regard, microglia stimulation has also been hypothesized to be helpful for the initial stages of neurodegeneration, when microglia cells tend to remove α-synuclein of the external area resulting in neuronal discharges or induced apoptosis leading to neurodegeneration^32^. Long-term stimulation of microglial, on the other hand, dramatically raises the quantities of pro-inflammatory cytokine and Reactive Oxygen Species (ROS), resulting in a worsening neuronal death scenario^32^. It is also known that α-synuclein buildup causes oxidative damage via two adjacent trajectories: actively promoting the formation of excess ROS or indirectly interacting with the scavenging of defective mitochondria from which the bulk of ROS are formed^33^.

Normal humans have systems in place that prohibit proteins from misfolding and accumulating inappropriately. These proteins, for example, are collected by the lysosomal and ubiquitin-proteasome systems, and specific chaperones could rectify such misfolding aggregates^34^.

#### Ubiquitin-Proteasome System

Among eukaryotes, ubiquitin is a 76-amino-acid constituent that is bonded covalently to target specific peptide complexes for disintegration through the ubiquitin-proteasome system (UPS). Approximately three decades ago, the existence of ubiquitin-immunopositive LBs inside the cerebellum of patients with PD was discovered^35^. Proteasome dysfunction was eventually discovered inside the ventral striatum of PD patients^36^. Notably, biological research demonstrates that UPS disorder plays a role in PD pathology. Similarly, PD-associated gene variability on PARK2^37^ and UCHL, which encapsulate the ubiquitin E3 ligase parkin and the deubiquitinating enzyme UCHL1, respectively, have also been identified^37^. Among healthy tissues, the UPS represents highly effective cellular clearance machinery, even though it is primarily accountable for the breakdown of shorter polypeptides into tiny cytoplasmic and plasma membrane peptides^36^. In the cytosol, nuclei, and endoplasm, it is deemed essential in the destruction of unfolded peptides or defective peptides^38^. In the etiology of PD, disruption or breakdown of this essential biological machinery has resulted in the accumulation of unfolded amyloid proteins like LB as well as an upsurge in neurotoxicity within SNpc^38^. Numerous additional proteins, including parkin and UCH-L1, as well as UPS, are implicated in the breakdown of misfolded α-synuclein during PD^38^. UCH-L1 is implicated in the synthesis of ubiquitin, which is co-localized with LB^38^.

#### Lysosomal autophagy pathway

Autophagy, which comprises macro-, micro-, and chaperone-mediated autophagy (CMA), are specialized systems that act as alternate peptide clearing machines within each cell, allowing LBs to be degraded in PD^38,39^. Particularly under relaxing circumstances, micro-autophagy is primarily involved in the destruction of tiny cytoplasmic proteins, whereas macro-autophagy is accountable for the breakdown of larger complexes. CMA is more specific since it interacts with heat-shock cognate protein (HSC70), which binds to tiny soluble proteins and degrades them via a unique pentapeptide targeting motif (KFERQ)^38^. Several lysosome and autophagy-related elements are dysfunctional or differently regulated in PD, analogous to results with the UPS mechanism. The autophagosome marker LC3-II was found to be elevated in substantia nigra neurons of the PD brain, indicating aggregation of autophagic vacuoles^40^. In other assessments, however, key lysosomal proteins (LAMP1 and LAMP2A) and various heat shock protein family molecular chaperones (such as HSC70 and HSP35) were discovered to be diminished^40^. The α-synuclein has just been demonstrated to be preferentially translocated towards lysosomes, where it is degraded by the CMA^38^. As a result, CMA malfunction reduces the effectiveness of α-synuclein degradation, resulting in an overabundance of this protein and a considerable reduction in neurotransmission^38^.

#### Protein and membrane trafficking mechanism

Cell membrane trafficking is also a type of intracellular transportation wherein larger components within transportation vesicles seem to be capable of reaching their intended locations despite trying to cross membranes^38,41^. As a result, membrane trafficking is important not only for regulating cell equilibrium but also for meeting unique needs throughout growth, differentiating, signaling interpretation, and transmission. Improper endocytosis trafficking, comprising mutations or aberrant production of key endocytosis elements, are also significantly linked to PD^42^. This includes the retromer subunit VPS35 and the retromer-associated protein receptor-mediated endocytosis 8 (RME-8)^43^, auxilin^41^, and synaptojanin^41^. Endocytosis trafficking, like AD, can cause PD disease in a variety of ways, including impacting pathogenic α-synuclein aggregate intake, modifying synaptic vesicles or neurotransmitter receptors traffic, and disrupting lysosome equilibrium and autophagy^41^. Disorders in various trafficking pathways may cause PD pathogenesis. LRRK2, which is disrupted in 1% of sporadic and 5% of familial PD cases, is a finding of recent studies^41^. LRRK2 phosphorylates numerous RAB GTPases across multiple trafficked stages^44^, while more prevalent PD mutations activate LRRK2^41^. Excess LRRK2-mediated activation impairs these RABs’ capacity to interact with transcription elements and functional molecules, causing traffic disruption^44^. RAB29 increases LRRK2 activity at cellular membranes and is associated with PD independently, implying that such protein (co)operates in a similar disease mechanism^41^.

### Mechanism 2: neuroinflammation

Another mode of PD pathogenesis is neuroinflammation (Fig. 3). The presence of a severe inflammatory response within the brain is mostly regulated by localized astrocytes. Although the presence of astrocytes and microglia have been increasingly identified in PD, it has remained a less explored area to date. Microglia and astrocytes generally seem to be engaged in the clearing of external material, that may help neurons endure. Active microglia can produce neurotrophic substances like brain-derived neurotrophic factor and glial-derived neurotrophic factor, but they might also produce damaging reactive oxygen and nitrogen species (ROS and NOS) and pro-inflammatory cytokines thereby leading to PD^7^. In PD patients, various innate and adaptive immune response aberrations have been identified, along with an upsurge in proinflammatory cytokines and a modified intracellular community (such as monocytes and their precursors)^31^. Clinically randomized trials indicate a connection between autoimmune illnesses and PD. An indication of inflammatory cellular activity (such as microglia) on molecular neuroimaging and neuroinflammation characteristics in experimental PD models has shown a confirmation of this mechanism.

### Mechanism 3: neuronal abnormality

Another alternate pathway of PD pathogenesis involves loss of dopaminergic neurons in the substantial nigra-striatum in the patients’ brain^16^. The 1-methyl-4-phenyl-1,2,3,6-tetrahydropyridine (MPTP)-model of PD exhibited an increase in expression of specific connexins (discussed in detail in two separate sections below) in the striatum, although the coupling of astrocytes was not increased^13^. Change or malfunction of gap-junctions in the brains of PD patients have not been reported yet thereby its pathological role in PD is ambiguous so far. Therefore, the mechanism and significance of gap-junctions in PD pathogenesis have been explored in detail in this article. Although most of the mechanisms of tremors and dyskinesis which are commonly observed in PD patients are still obscure, the inferior olive has been focused on as the pathological generator of tremors^14,15^. Neurons of the inferior olive are electrically coupled through gap-junctions which play a role in creating oscillatory activity. Some studies have reported that the GJIC of inferior olive neurons is responsible for tremors^13,14^. All of these play a major role in the spreading of PD. These significant hallmarks are frequently correlated with several other interconnected events, such as vesicles transit interruption, the decline of tubulin integrity, nerve cell inflammation, trophic factor disruption, iron metabolic pathway dysregulation, endoplasmic reticulum impairment, poly (ADP-ribose) polymerase, and other enzymatic activation, to name a few^31^.

### Mechanism 4: Mitochondrial malfunction and mitophagy

Mitochondria are the “powerhouses” of cells, producing adenosine triphosphate (ATP) via oxidative phosphorylation^45^. ROS are produced by the electron transport chain during the ATP generation process. Radicals from complex I are transferred to the mitochondrial matrix, but radicals from complex III are delivered to both the inner membrane space (IMS) and the mitochondrial matrix^33^. Reduced mitochondrial complex I activity is also reported in PD patients, additionally, the utilization of its inhibitor (e.g., rotenone) is believed to trigger mitochondrial destruction (such as reduced mitochondrial prospects, cytochrome C release, initiation of the caspases, and eventual induction of apoptosis) in exploratory PD models^31^. Mitochondrial malfunction increases oxidative stress and ROS generation; which in turn, are damaging to the electron transport system, contributing to more ROS production^33^. It was proposed that mitochondria-induced ROS overconsumption plays a significant role in apoptosis and the development of delayed neurological diseases, notably in idiopathic PD^46^. Throughout the cerebral regions of PD patients, there is significant lipid peroxidation, glutathione deprivation, and an elevation in protein oxidation^34^. Dopaminergic oxidation results in the production of serotonin quinone, which can significantly affect proteins. Mitochondrial failure causes a lack of ATP, which is required for dopaminergic neurons to transmit electric impulses, retain ionic gradients, and release dopamine^33^. All these can lead to PD pathogenesis.

### Mechanism 5: gut-brain microbiome interaction

There is an increasing amount of information that reveals the gut-brain connection plays a role in PD etiology, with the vagus nerves acting as an “expressway” for accumulated α-synuclein to go from the intestinal system to the lower brainstem (Fig. 4)^47^. Some reports show that cells obtained from PD patients that were implanted into recombinant α-synuclein expressing rats caused motor impairments, and antibiotics therapy restored some of the problems, whereas microbial recolonization exacerbated the pathogenesis^48^. Moreover, numerous investigations have indicated that vagotomy and appendectomy may lower the chance of acquiring PD. However, more research is required to fully understand the involvement of the gut tract and microbiomes, infections, and inflammatory responses in causing α-synuclein aggregate and spreading to the nervous system as a pathological process for PD.

## Why is it important to study gap-junctions and ion-channels in the context of PD?

Gap-junction (GJ) channels and associated connexins (Cxs) are complicated proteins that play critical roles in the nervous system and neural cellular interaction medium. Neurons, astrocytes, oligodendrocytes, and microglial cells express a diverse set of Cxs which are required during intercellular interaction and the transport of small molecules, ions, as well as neuronal and glial transmitters^17^. They not only play an important function in the CNS but also play a pathological role in many neurodegenerative diseases like PD, AD, and HD^49^. However, their exact role and mechanism of action in the case of PD are scarcely explored so far, which otherwise is very important to not only understand the disease pathology but also to develop a treatment for it. Since PD is associated with the development of astrocytosis and gliosis, the elevation in connexin synthesis in astrocytes and microglial cells is expected^49^. The most common connexin variation in inflammation circumstances, i.e. Cx43, is reported to be elevated in PD. The production of inflammation chemicals by activated microglia disrupts capillaries and produces extremely unstable vessels. Furthermore, active microglia enhance connexin hemichannel activation, culminating in a bilateral flow among the external and internal cellular compartments^50^. Thus, it is clear that different Cxs play different roles in connection to PD as discussed in detail in a separate section below which forms the origin of writing the current review article to explore gap-junctions in terms of their mechanism of action and plausible use of them as targets for therapeutic interventions in PD.

Similarly, multiple ion-channels work together to control the secretion of dopaminergic as well as the firing action of substantia nigra neurons. The abnormal transport of different ions within the internal environment is caused by the deregulation of these systems. This disrupts intracellular signaling pathways, cellular equilibrium, and energy metabolism. This indicates that ion-channels also play a critical role in the elevated sensitivity of dopaminergic neurons to degeneration in PD. Blocking ion-channels is also an appealing mechanistic way to combat the progression of neurotoxicity and thus has been explained in detail in yet another section below.

## Different types of gap-junctions: their mechanism of action and functions in relation to PD

**Fig. 5:**
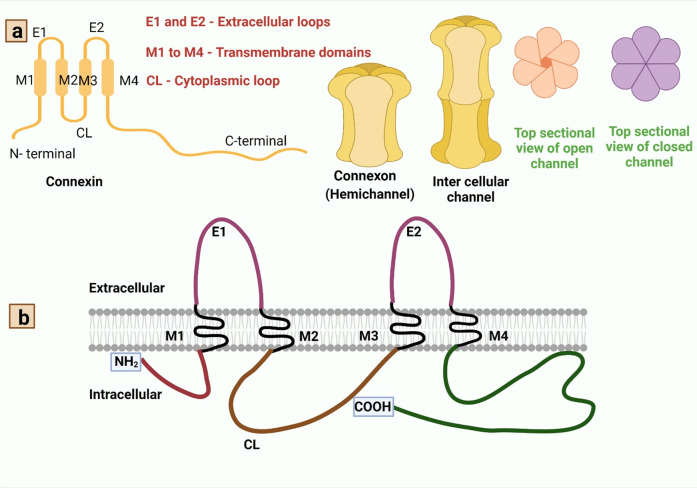
Structural organization of connexins and connexons to form gap-junction. Panel a: Gap-junctions are made up of homomeric or heteromeric hexamers of connexin, connexon (hemichannels) that are situated side by side in the plasma membranes of two neighboring cells^51^. Connexins have four strongly organized transmembrane segments (TMSs), with predominantly unstructured C and N cytosolic termini, a cytoplasmic loop (CL), and two extracellular loops (EL-1) and (EL-2). Connexins create hemichannels, or connexons, within clusters of six, and two hemichannels join to produce gap-junctions. Panel b: The connexin polypeptide crosses across the membranes four times (black), exposing its amino-terminal and carboxy-terminal regions to the cytosol and connecting via two extracellular and one intracellularly exposed loops^208^. The extremely consistent amino-terminal domain (maroon) regulates its channeling pore whereas the two invariant disulfide-linked external loops (magenta) regulate hemichannel coupling among neighboring channels^209^. The length of the cytoplasmic loop (brown) differs widely amongst connexin subclasses^210^. Within the connexin familial member, the span of the cytoplasm terminal (deep green) and its amino acid composition are highly variable^211^.

Gap-junctions (GJs) have complicated arrangements made up of homomeric or heteromeric hexamers of connexin (Cx), connexons (hemichannels (HCs)) that are situated side by side of plasma membranes of two neighboring cells^51^. Connexins create hemichannels, or connexons, within clusters of six, and two hemichannels join to produce gap-junctions. Each solitary GJ channel consists of two opposed hemichannels termed connexons, and are typically comprised of six proteins called connexins^52^ as depicted in Fig. 5. Different cell types in the mammalian brain express at least ten different types of connexion which in turn makes the organism to be diversified and forms complex intercellular communications. Connexins, one on either side, could be classified under subclasses α, β, γ, δ, ε, depending on the quantity of homology and the extent of the cytoplasmic loop as mentioned in Fig. 5 and Table 1. Astrocyte expresses: Cx43, Cx30, Cx26; oligodendrocyte expresses: Cx32, Cx29, Cx47; microglia express: Cx43, Cx32, Cx36; and all endocytic cells express three connexins: Cx37, Cx40, and Cx43^52^.

**Table 1:** Types of various connexins expressed in distinct subgroups of gap-junctions (^212,213^).

Cellular/Gap-Junctions	Connexin	Subgroups
Astrocytes	Cx26	β
	Cx30	β
	Cx40	α
	Cx43	α
	Cx45	γ
Oligodendrocytes	Cx29	n.a.
	Cx32	β
	Cx36	δ
	Cx37	α
	Cx40	α
	Cx43	α
	Cx45	γ
	Cx47	γ
Neuronal Precursors	Cx26	β
	Cx33	α
	Cx40	α
	Cx43	α
	Cx30.3	β
	Cx31	β
	Cx46	α
	Cx50	α
	Cx57	α

*n.a.= not available.

Cx37 and Cx45, like Cx43, are substantially upregulated in PD, but Cx26 and Cx25 are negatively regulated^49^. In PD, increased production of Cx37, Cx43, and Cx45 in cerebral endothelial cells has been linked to capillary leaking and improved endothelial cell interaction^49^. Cx47 and Cx32 are dysregulated in PD, although Cx30.2 stays unaltered. In neurological illness, these three oligodendrocyte connexins were associated with neuronal damage^49^. Cx31.9, a protein generated in the cerebrum, was also discovered to be deregulated in PD patients^49^.

### Homotypic Gap-Junctions

#### Between neurons

So far, eight distinct Cxs were identified in specific neuron cell lines and examined ex vivo and in vivo in developing animal models. As seen in Table 1, the majority of these Cxs are present in a wide range of cells and therefore are not confined only to neuronal cells only. Several investigations have shown that neurons express Cx32 and Cx43 (Table 1). Cx36 expression is constrained to neuronal, according to the latest survey whereas Cx32 and Cx43 had been identified in oligodendrocytes and astrocytes, correspondingly^53^. Cx36 is the first Cx variant to be generated solely in CNS nerve cells and it has unusual functioning features in addition to other Cxs^54^.

#### Between astrocytes

Astrocytes have a syncytium arrangement because of their extensive abundance with tight junctions which allows for rapid interstitial transport of molecules and transmitting elements. Intracellular interactions within cultivated astrocytes are regulated via tight junctions constituted predominantly of Cx43. Consequently, modest quantities of Cx30, Cx40, and Cx45 had been observed^55–57^. Cx43 production, as well as linkage efficacy, differs among culture astrocytes, generated from various cerebral regions, for example, being modest among mouse striatum and high in the hypothalamus region of astrocytes^58^. Such variations might point to a diverse localization of Cx43 in the cerebrum. Furthermore, evidence suggests that the expression of Cx30 is more in the adult gray matter of astrocytes, while the respective amounts of Cx43 and Cx30 fluctuate depending upon the stage of development as well as the site examined^59^. Cx26 previously assumed to exist only aberrantly inside cerebral parenchyma has lately been detected within cellular/gap-junctions of astrocytes^60^.

#### Oligodendrocytes

In vivo and ex vivo studies suggest that there are expressions of connexin genes Cx32 and Cx45 in oligodendrocytes. Cx29, a recently identified connexin, has been found in oligodendrocytes. Various immunofluorescent studies indicate that newly generated connexin Cx45 was expressed in oligodendrocytes^61–63^.

#### Microglial, ependymal, and meningeal cells

Lately, the expression of connexins within microglia cell lines was revealed, and modest amounts of Cx43 were reported. Cx43 expression gets elevated after microglia cells get activated. Cx43 is located just at boundaries among activated glial cells and also they get coupled with dye with gap-junctions in the subsequent situation^64^. Cx26 and Cx43 had also been reported in epithelial cells (ependymal cells) of ventricles in cerebrum^65,66^. Gap-junctions are prevalent across the growing and mature meninges. In meningeal cells, the three connexins which have elevated expressions are Cx26, Cx30, and Cx43^60,67^.

### Heterotypic Gap-Junctions

#### Within microglial cell lines

Although primary Cx reported is Cx43 in astrocytes and Cx32 in oligodendrocytes, which are probable donors to both sides of its channels. Conversely, Cx43 and Cx45 could indeed communicate together and may generate heterogeneous linkages^68^.

#### Between glia and neurons

Here, the identity of connexin implicated throughout this heterotypic junction is unknown. Expression of connexin in glial cells are Cx26, Cx32, and Cx43, and the expressions of connexins in neuron are Cx26 and Cx32. An investigation of more than 5000 connections comprised of various Cxs (i.e., Cx30, Cx32, Cx36, and Cx43) from numerous cerebral areas revealed tight channels between astrocytes and neurons^69^.

#### Connexin Hemichannel

Under standard conditions, Cx hemichannels need not be active. It stops particles and relatively small compounds from entering the cell and also cytoplasm ingredient spillage, which might lead to cytoplasm deficiency of important molecules. Cx43 hemichannels may activate within cerebral astrocytes following membrane disruption exposition towards a reduced calcium ionic medium, or metabolism limitation. Immune cells may be able to save deceased ones through exchanging molecules as well as critical compounds across complete Cx43 cell signaling junctions.

## What is known about the role of gap-junctions in the spread of PD pathology?

**Fig. 6:**
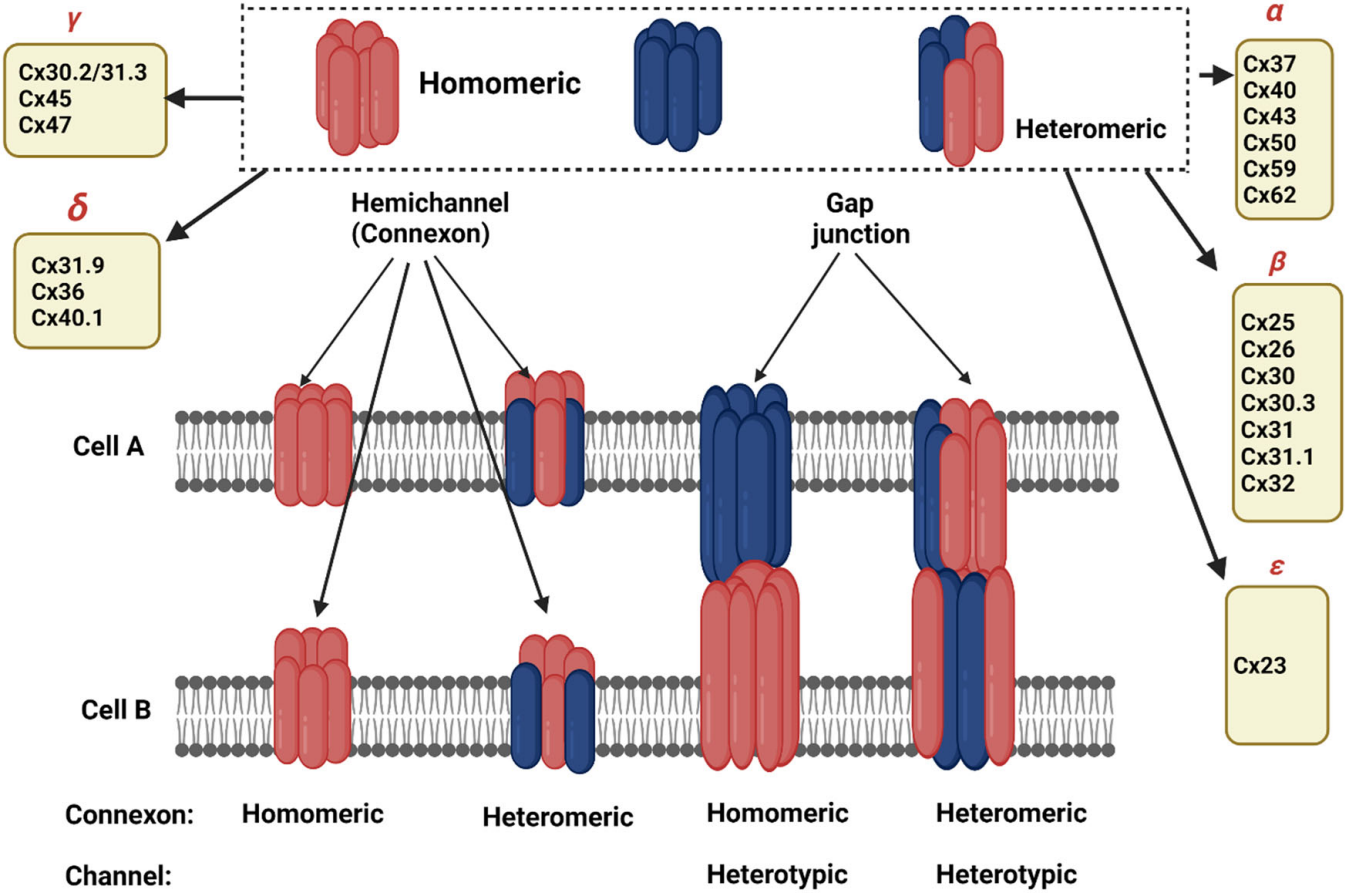
Pictorial depiction showing that connexins can be arranged in many different ways in a gap-junction channel. The diagram depicts several constituents of the gap-junction channel. A particular kind of connexin forms homomeric connexons. Heteromeric connexons are made up of greater than a single connexin kind. Whenever connexons of a similar structure create a gap-junction channel, it is referred to be a homotypic channel. Whenever the constituents of the connexons vary, the channels are known as heterotypic. Depending on homology, the connexin group is classified into five subgroups (α, β, γ, δ, and ε)^211^. Connexins oligomerize into homomeric or heteromeric hexamers alongside adjacent connexins of a similar subtype as well as on rare occasions, with connexins of different subtypes, resulting in a very diverse channel configurations^211^.

**Fig. 7:**
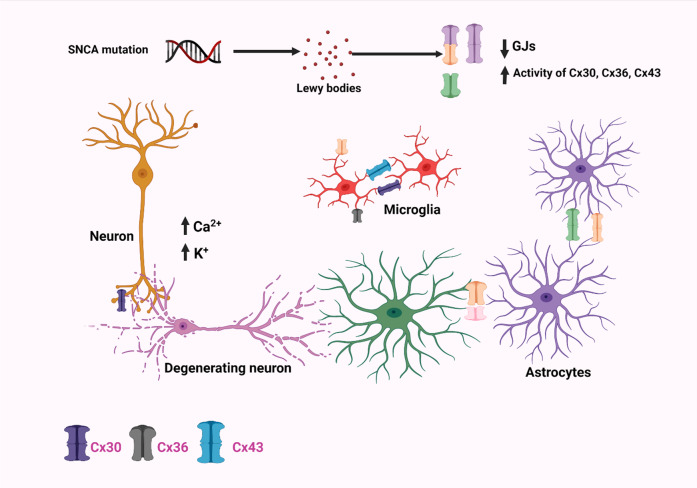
Pictorial depiction of major changes occurring in the regulation of Cx30, Cx36, and Cx43 in neurons, astrocytes, and microglia, respectively in a PD patient. SNCA mutation leads to the accumulation of Lewy bodies which in turn increases the opening of Cx43 HCs, thereby resulting in elevated internalized Ca^2+^ levels and cytokine production. SNCA: Synuclein Alpha gene.

Although all Cxs are crucial for intercellular signaling inside the brains, recent research has revealed that Cx30, Cx36, and Cx43 play key functions in cellular regulation, neurodegenerative processes, and neuroprotective properties. Cx30, one of the major Cxs found in astrocytes, plays an important function during astrocyte-astrocyte communications and nutrition transportation^70^. Moreover, α-synuclein has been shown to increase the production of Cx43 HCs (hemichannels) in cortical astrocytes, resulting in an increased flow of calcium ions in addition to other effects like activating cytokines, cyclooxygenase 2, and inducible nitric oxide synthase as shown in Fig. 6^71^. Rodent models of PD were experimentally utilized to determine if Cx-mediated HCs perform a critical function for the astrocyte malfunction. The most common neurotoxin chemicals which cause degeneration mechanistically modulate Cx43 HCs include 6-hydroxydopamine (6-OHDA), 1-methyl-4-phenyl-1,2,3,6-tetrahydropyridine (MPTP), and rotenone^72^. MPTP produces a rapid, although temporary rise in the striatum Cx43 mRNA, which is accompanied by a persistent elevation in Cx43 immunoreactive puncta^73^. Likewise, using an in vitro and in vivo rotenone-induced model of PD, higher levels of Cx43 were found alongside elevated GJIC, which was also again confirmed within a mouse PD model with an observed rise in phosphorylated Cx43 which is required for GJIC^72^. The elevated level of phosphorylated Cx43 was observed in Striatum and Hippocampus regions. All these studies have shown an increase in GJIC with an increment in Cx43 level in astrocytes^72^. Such a surge in Cx43 overexpression resulted in higher HC functioning, improved GJ interaction, plus heightened cytoplasmic Ca^2+^ concentrations, all of which contribute to neurotoxicity and neurodegeneration^30^. Other experiments employing rotenone demonstrated a downregulation of Cx43 resulting in decreased GJ porosity in primary culture astrocytes, indicating that astrocytic GJ malfunctioning might be involved in underlying PD pathogenesis^74^. Moreover, studies involving 6-OHDA-induced mouse models of PD resulted in significant levels of Cx30, but hardly Cx43, throughout the stria; however, levels of Cx43 and Cx30 have significantly increased surrounding arteries, indicating enhanced metabolic interaction^75^. Cx30 and Cx43 were found to be increased inside the stria of an acute PD animal model induced by MPTP treatment. Furthermore, MPTP therapy has been demonstrated in a Cx30 KO animal model to increase the overall depletion of dopaminergic neurons and downregulate the S100a10 genes, which is crucial for cell motility, cell to cell translocation. This in turn represses GFAP and astrogliosis within the striatum^76^. These findings imply that Cx30 insufficiency reduces the neuroprotective role of astrocytes within stria and thus enhancing Cx30 functions which is recommended as a treatment option for PD patients^30^. Cx36 GJ channels vary from other Cx variants in terms of regulation characteristics, like reduced unified conductivity as well as susceptibility to transjunctional voltages; alterations related with any of these has a regulatory roles in PD^77^. GJs were subsequently suggested to be implicated in the internal process of synchronizing, owing to a potential GJ reconfiguration and GJ connectivity linked to dopamine levels, wherein GJ conductivity decreases as dopamine levels increases as shown in Fig. 7.

Altogether, these findings demonstrate the potential impacts of Cx30, Cx36, and Cx43 on PD and in the foreseeable future, it might be useful to focus on these gap-junction regulations in addition to chemical therapies for improving neurocognitive impairments in PD patients.

## Different types of ion-channels, their mechanism of action, and their functions concerning PD

**Fig. 8:**
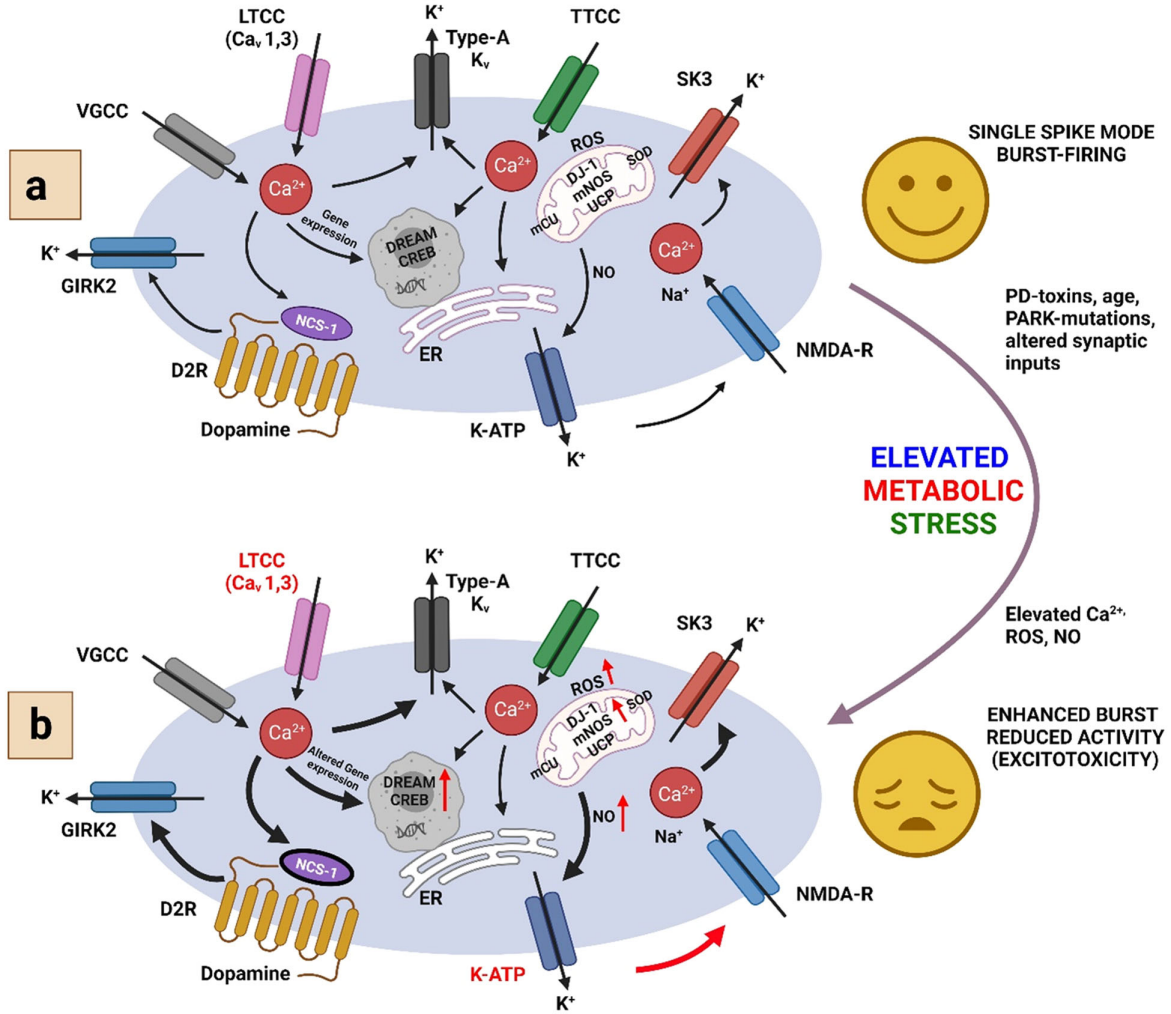
Pictorial depiction of different types of ion-channels in the CNS and their mechanism of action under unstressed (Panel a) and stressed (Panel b) conditions. Panel a: Mechanistic view of networking of ion-channels in SN dopamine nerve cells in PD and other health states. Under physiological conditions, activity-dependent Ca^2+^ loads due to (1) active participation of LTCC (L-type Calcium Chanel) and VGCC (Voltage-Gated Calcium Channel), with the help of KATP/NMDA-R, which mediates bursting activity and Ca^2+^-stimulated TCA (Tri Carboxylic Acid) cycle and m-NOS, (2) generated ROS for SN DA, which is controlled by ER (Endoplasmic Reticulum), mitochondrial Ca^2+^-buffering, UCP, ion-channels that reduces electrical activities like D2-AR/NCS1/GIRK-2, KATP, SK-3, anti-oxidative enzyme kinetics such as DJ1, SOD (Superoxide dismutase) function, and (3) Ca^2+^ dependent gene expression. Panel b: As the relative level of Ca^2+^, ROS, NO (Nitric Oxide), and metabolic stress increases, the controlled mechanism may not be enough for altered SN dopamine activity, altered gene expression to remain under physiological regulation thereby leading to apoptosis. TTCC: T-type calcium channel, DJ-1: Mutations in *DJ-1* (PARK7), D2R: Dopamine Receptor, ROS: Reactive Oxygen Species, GIRK-2: G-protein-coupled inwardly rectifying potassium (GIRK) channel.

Dopaminergic Neurons (DA) form the bilateral dopaminergic pathway which is identified by their ability to transfer current from irregular tone or the activity played by the pacemaker to the bursting out of the activity within or outside an organism^78^. Ion-channels control their firing action potential and the pivot-like activity of dopamine, released from the axonal terminus of subcortical basal ganglia, functions like a motor^18,79^. Positively charged potassium is responsible for maintaining a balance and equilibrium within the cell thereby maintaining its volume and at the same time maintaining the potential of the cellular membrane^18,79^ (Fig. 8). Depending on the sequences of amino acids, K^+^ channels can be classified into three major classes: (1) voltage-gated K^+^ (Kv) channels, (2) Kir channels which rectify the inward activities such as Kir1 − 7 and KATP channels, (3) K2P channels with two pores such as K2P15–K2P18, K2P9–K2P10, KCNK, K2P12–K2P13, and K2P1–K2P7. For their vast roles in neuronal activities, potassium ions are considered one of the important ions in PD.

### Voltage-Gated K^+^ (Kv) channels

These channels are responsible for the efflux of K^+^ and control the potential of the cell membrane via a change of voltage. Kv channel tetramers are composed of homomers and heteromers of four subunits, which comprise Hexa-transmembrane segments (S1-S6). Interaction of Kv4.3 with K-Chip3 generates type AK^+^ currents, which inactivates the components of the extracellular K^+^ currents located in SNc dopaminergic nerves. Type AK^+^ channels in dopaminergic nerves of substantia nigra contribute to pacemaker-controlling tonic activities^80,81^. Type-AK^+^ channel in mesencephalon dopaminergic nerve cells activates mitogen-activated kinases, thereby suggesting type-AK^+^ channels to be a promising diagnostic marker in Parkinsonism^80,82^.

### Delayed rectifier K^+^ channels

Delayed rectifier K^+^ channels are a group of slow opening and closing voltage-gated K^+^ channels, including Kv1.1–1.6, Kv2.1–2.2, and Kv3.1–3.3^80,83^. The increase in the expression of surface Kv2.1 channels led to the downfall of DA in the striatum terminals of DA and SN^84^. When the Kv2.1 channels were inserted into the cellular membranes, it led to a decrease in the concentration of K^+^ activating enzymes like nucleases and proteases intracellularly, leading to cell death^85^. With this increase, the intracellular concentration of Ca^2+^ increases, thus promoting the influx of Ca^2+^ into the cell, leading to the damage of the cell and thereby linking with the flow of K^+^ inside the cell, hence causing diseases associated with CNS^86^. The projections from the medium spiny neurons of the caudate and putamen to the internal segment of the globus pallidus and substantia nigra pars reticulata are part of a “direct pathway”. The GP is an interconnection of all major parts of the microcircuit of the basal ganglia. Abnormal discharge of high-frequency leads to stiffness and tremors in PD^87^. The DA nerves in PD gets degenerate during the inflammatory process of the basal ganglion. The *Stichodactyla helianthus* K^+^ channel acts as a blocking toxin for the Kv1.3 channels, which suppresses microglial cell and T-lymphocyte activation in PD^88^. The current generated by K^+^ mediates Kv1.3 channel depolarization via robust negative feedback, thus proscribing SCIN sencitability^89^. In the striatum of PD mice, acetylcholine release from cholinergic interneurons decreases the excitability of Kv1.3 current inhibition, thereby leading to the prevention of change in acetylcholine concentration in the striatum^90^.

### ERG K^+^ channels

Kv11 or ERG K^+^ channels belong to the family of Kv channels which encodes three different genes e.g., eag, elk, and erg genes^91^. This channel modulates the functioning of STN neurons. Recent developments showed that the inhibitors of ERG channels reduce STN neuron burst discharge and improper functioning in PD mice, likely E-4031 and dofetilide, whereas PD-118057 acts vice-versa^92^. The malfunctioning of STN neurons is associated with the change in neuropsychiatry, and antipsychotics partly block the ERG channel inside nerves, thus affecting the cells. Therefore, these channels provide unique methods for PD treatment and treatment of other neurodegenerative disorders^93^.

### GIRK channels

GIRK channel is a part of the vast family of channels, especially Kir1–7. These types of channels mediate the synaptic action of a large number of nerves in the CNS, which shows pivotal activity after the inhibition of synapses is completed. Four genes were found in mammals that were structurally similar: GIRK1, GIRK2, GIRK3, and GIRK4^94^. Hippocampus is that region of the brain where the GIRK2 is majorly expressed, followed by the amygdala and Substancia nigra^95^. The Purkinje cells and VP (ventral pallidum) neurons are some examples where the GIRK4 channels are less populated^95^. Auto somatodendritic D2 receptor activates the GIRK channels^96^.

### KATP channels

KATP channels comprise of two basic units which differ structurally, like Kir6.1 − 6.2, which is the pore-forming unit of the Kir6 channel, and sulfonyl-urea receptors, which include SUR1, SUR2, SUR2A, and SUR2B. These channels are responsible for the identification of measure of energy units in the cellular components, thereby forming a combination of membrane excitation and metabolism of the cell^97^. The effects of oxidative stress, mitochondrial damage, and decreased ATP concentration in the DA nerves of SN, leads to the activation of the channel and hyperpolarization of the cellular membrane thus protecting the CNS by reducing excitability in the nerves. Studies have shown that the Kir6.2/SUR1 channel has been functionally expressed by SN DA neurons and the VTA. However, a selective increment was observed in SUR1 expression in the Substance nigra of MPTP mice model of PD^97^. It was also observed in rodents and some cases of PD that high levels of UCP, present in the inner lining of mitochondria express VTA DA neurons, and even a minute uncoupling can result in the reduction of ROS formation thereby decreasing the opening of KATP channel^98^. Lower concentrations of UCP-2 in SN DA neurons showed that the channels remain sensitive to metabolism and thus the possibility of KATP channels remaining open is highly increased^97^.

### K2P channels

Identified in the name of “leakage” or “background” channels, the K2P channels depend on K^+^ level outside the cell. It has four transmembrane domains and two P regions^99^. A vital K2P channel functional in the CNS is TREK-1^100^, which maintains an imbalance of electrical charges between the interior of electrically excitable neurons, strongly influencing the outward movement of current and maintaining the propensity of neurons by electric charge shift^101^. TREK-1 was shown to have been activated by riluzole, whose characteristics are similar to NDMA receptors, attenuates L-DOPA induced unusual involuntary movement in rats, which is further reduced by CREB1 induced L-DOPA receptors^102^. Low-intensity pulsed ultrasound prevents or delays MPP^+^ induced DA neurons are degenerated by low-intensity ultrasound, K2P channel activation, and downward movement of ion-channels^103^.

### SK channels

SK channel is a complex macromolecule that forms an important pore and gets activated with the increased level of Ca^2+^ in the cytoplasm. SK channel forms a four-membered family from SK1–4^104^. SK1–2 express themselves in the brain tissues that can alter the membrane potential, especially the cortex and hippocampus. SK3 express themselves in the tissues of secondary concern or sub-cortical areas, like the locus coeruleus, dorsal raphe, and SN, whereas SK4 channels occur in tissues of the heart, lungs, and placenta^105^. Constitutive binding of calmodulin to the closer proximity of C-terminus detects the action of Ca^2+^. Phosphorylated calmodulin influences susceptibility of SK channel. Calmodulin after binging to Ca^2+^, activates SK channels leading to a change in the membrane potential of a cell, thereby reducing its excitation^105^ (Fig. 8).

### Voltage-Gated Ca^2+^ channels

Voltage-gated Ca^2+^ channel (VG-Ca^2+^channel) forms the main components of hyperpolarization. These members of VG-Ca^2+^ channels play an important role in cell signaling. CaV1 sub-member of this family of Ca^2+^-channels influences cells to contract, secrete, regulate expression of genes, integrate, and transmit synapses in nerve cells. The next member in this family is CaV2 which initiates neuron communication with a target cell across fast synapses. The third sub-member is CaV3 which is important for sending the electrical signal down the axon, thalamic neuron, and muscle cells of the heart^106^.

## What is known about the role of ion-channels in the spread of PD pathology?

### Roles of Kv-7/KCNQ channels

Membrane currents are the subliminal voltage-gated K^+^ currents originating from the Kv-7/K-CNQ channel. The K-CNQ families form a Penta-member of K-CNQ1–K-CNQ5 and Kv-7.1–Kv-7.5, whose expression is high in the CNS and PNS. K-CNQ2 and K-CNQ4 confine specific areas of SNs and VTA (Ventral Tegmental Area) of the mesencephalon^107,108^. Current modulates the firing frequency of dopaminergic nerves in the midbrain. K-CNQ channel activators induce hyper-polarization of dopaminergic nerve cells and inhibit synaptically induced excitation. Kv-7 channels are positioned at both presynaptic and postsynaptic sites of the striatum, counteracting the high excitatory inhibition of dopamine-D2 receptors^109^. XE-991 enhanced supraliminal synapses and depolarizes the projected nerves of GABA in the striatum^110^. XE-991was administered into the SNc, which reduced catalepsy obtained by haloperidol insertion^111^. XE-991 also contains protective abilities against 6-OHDA induced degradation of SN striatum. It was proven that the SK channel, Type-A K^+^ channel, and Kv-7/K-CNQ channel form the important substrate for PD diagnosis. Blockers/activators of the three K^+^ channel protects DA nerve cells in SNc, modulates neuron excitation, influences release of DA, and attenuates motor-symptoms. K-CNQ channel-opener, retigabine, downregulated L-DOPA induced dyskinesia in 6-OHDA lesioned rodents. Increased ability of GABA in the nerves of the striatum are required for the treatment of motor-fluctuation and dyskinesiasis, with the help of L-DOPA, thus reducing MSN-activity in the striatum^112^.

### Role of GIRK channels

GIRK2 influences the generation of GIRK current in nerves, thereby decreasing the excitation of nerves. D2 receptor proteins release the G-protein β-γ, when dopamine binds, thus keeping the GIRK2 channels open^113^. When the GIRK2 activates, it causes a change in the membrane potential of neurons inhibiting a negative response leading to a decrease in dopamine production. However, any sort of GIRK2 mutations in rodents could be the cause of gradual deterioration of the dopaminergic nerves in the Substancia nigra, leading to abnormal stabilization and tremor developments^114^. L-DOPA, a type of dopaminergic pro-drug is top-rated for PD diagnosis. Studies have shown that DOPA-quinones derived from 3,4-dihydroxyphenylalanine metabolism help in the association of α-synuclein protein, abnormality in functions of mitochondria, and protein degradation^115^. Synthesis of L-DOPA-quinone from 2-IBA is necessary for the stabilization of GIRK channels and counteracting neuron inhibition of SN DA nerves when they bind to the Cys residues of GIRK2 channels^115^.

### Roles of K-ATP channels

It has been observed that in MPP^+^ induced inhibitory action of mitochondrial respiratory chain complex-I, the SN DA neuron with the high level of Kir6.2/SUR1 channel expression leads to damaging of cells^116^, thereby partially forwarding towards apoptosis. Studies have shown that in the mid part of SN DA nerves associated with DMS, K-ATP channels promote changeover from sustained response due to tonicity to NMDAR mediated bursting within itself. KATP increases the Ca^2+^ concentration in nerves connected to NMDA receptor molecules^117^, thereby increasing the ROS generation which influences neuronal stimulatory action, further damaging thousands of neurons and causing induction of PD^118^. Removal of Kir6.2 promotes the accession of DA phenotypes from nuclear-receptor-related 1 precursor, inhibits a decrease in glial cell-line-derived neurotrophic-factor (NF), promotes cellular distinctions in the nerves of the midbrain, which harms microRNA133b concentration^119^, and inhibits an increase in Fe^2+^ concentration in the mesencephalon of MPTP treated mouse, thereby suppressing the regulation of IRP-IRE lighter chain models and hence keeping SN DA nerve cells protected against MPTP-attack^120^. Hence, it can be said that Kir6.2 has a vast role to play in the regulation of KATP/Kir6.2 channels and in the diagnosis of PD.

### Role of a type K-1 channels

**Fig. 9:**
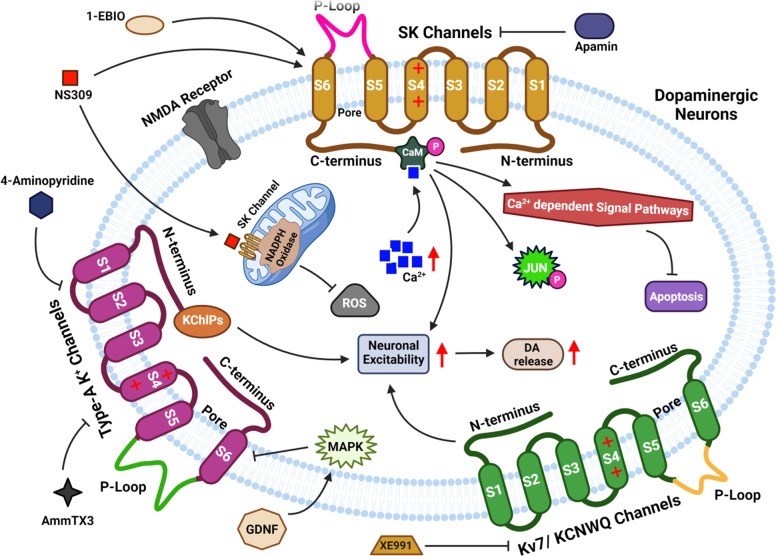
Mechanistic view of roles of different K^+^-channels in the diagnosis and treatment of the PD pathogenesis. Cellular and animal models of Parkinsonism showed that SK channels, Type-A K^+^ channels, and Kv-7/ KCNQ channels possess a potential for PD diagnosis. Activators and blockers of these three potassium channels protect the dopaminergic nerve cells in SNc, modulate excitation of nerve cells, influence the release of DA, and attenuate motor symptoms. NMDA N-methyl-D-aspartate receptor, NADPH Nicotinamide adenine dinucleotide phosphate oxidase, MAPK Mitogen-Activated Protein Kinase pathway, GDNF Glial-cell derived neurotrophic factor.

Kv-4 generated type AK^+^ currents which included tetrameric subunits with Hexa-transmembrane domains (S1–S4 and S5-S6) (Fig. 9). Among them, S5 and S6 domains are of type A subunit formed-loops in the Kv-4 channel, which selectively allow permeability of ions. S1, S2, S3, and S4 of type A subunits formed voltage sensor domains^121^. Auxiliary K-Chips co-assembled with the P-looped performers in Kv-4 subunits which forms a complex of K^+^ channels, which helped in the regulation, expressions, and gate-like characteristics of Kv-4 current. The Kv-4 contains three different genetic materials from Kv4.1–4.3. Kv4.2 and 4.3 express well in the mesencephalon of dopaminergic nerve cells^122^. Interaction of Kv4.3 with K-Chip3, generates type AK^+^ currents, which inactivates the components of the extracellular K^+^ currents located in SNc dopaminergic nerves. Type AK^+^ channels in dopaminergic nerves of SN contributed to pacemaker-controlling tonic activities^81^. Inhibitory action of type-AK^+^ channel depolarized and increased the excitation by changing over from tonic-firing to burst-firing, thereby influencing dopamine-release^123^. MSN integrates various signaling pathways inside the cell thus causing dynamic modulation of membrane excitation. Intrastriatal infusion of AmmTX-3 reduced the deficiency of motor neurons, reduced anxiousness, and restored memorization power in 6-OHDA treated rodents. Also, 4-AMP inhibited the type-AK^+^ channel, which decreased apomorphine-induced rotational-test (RT) in 6-OHDA-induced rodents. The glia-derived neurotrophic factor (NF), regulated developmental and functional parts of the CNS. Type-AK^+^ channel in mesencephalon dopaminergic nerve cells triggered mitogen-activated kinases, thereby suggesting type-AK^+^ channels to be a promising diagnostic measure in Parkinsonism^82^.

### Role of SK channels

SK channel regulates d*opaminergic neuron functioning* and excites the nerves of the basal ganglion. SN dopaminergic nerves are activated by these channels on induction of a single action potential by the offset of hyperpolarizing stimulus, reduction of a neuron’s firing rate, and maintaining the intrinsic current automaticity^124–126^. The boundary of the SK channel promotes N-methyl-D-aspartate-induced abnormal bursting in dopaminergic nerves, thereby activating Ca^2+^ dependent cellular activities and enhancing dopaminergic release in the corpus striatum, thus relieving PD symptoms. SK channel activation leads to neurotoxin-induced dopaminergic apoptosis (Table 1)^127^.

### Role of Voltage-Gated Ca^2+^ channels

Voltage gated calcium channels (Ca_V_) are responsible for exaggerating PD and blockade of the midbrain. However, when specific binders/blockers for each Ca_v_ isoform are absent, the exact role of either Ca_v_1.2 or Ca_v_1.3 in degeneration or protection of neurons is very difficult to understand. To decipher this mystry, Ca_V_ gene KO animal models were useful indicating that dihydropyridines have diverse effects on different types of these channel isoforms. Due to its exemplary roles in SN dopamine neurons and diverse spread across the brain Ca_V_ channels can be a potential drug target for PD, also to monitor the progress of any related treatment efficacy thereby reducing the side-effects of available therapeutics, if any (Fig. 8).

## The link between α-synuclein-GBA-LRRK2 and ion-channels/gap-junctions to develop PD

It has been discussed above in the introduction that there are seven known causation alleles for familial PD which include α-synuclein, GBA, and LRRK2^4,5^. Along with this α-synuclein-GBA-LRRK2 axis, within the complex neural network^9^, gap-junction-based synaptic vesicles remain scattered, thereby playing an important function in the developmental processes of the CNS and PD pathology. Moreover, gap-junctions and ion-channels both facilitate cell-cell interaction as well as the exchange of ions and tiny signaling chemicals^9^. They also act as a linker between the nerve cells and glia, namely astrocytes, microglia, and oligodendrocytes. Therefore, many structural or functional alterations in these junctions and ion-channels may lead to PD pathogenesis facilitated by α-synuclein-GBA-LRRK2 axis^10^.

**Fig. 10:**
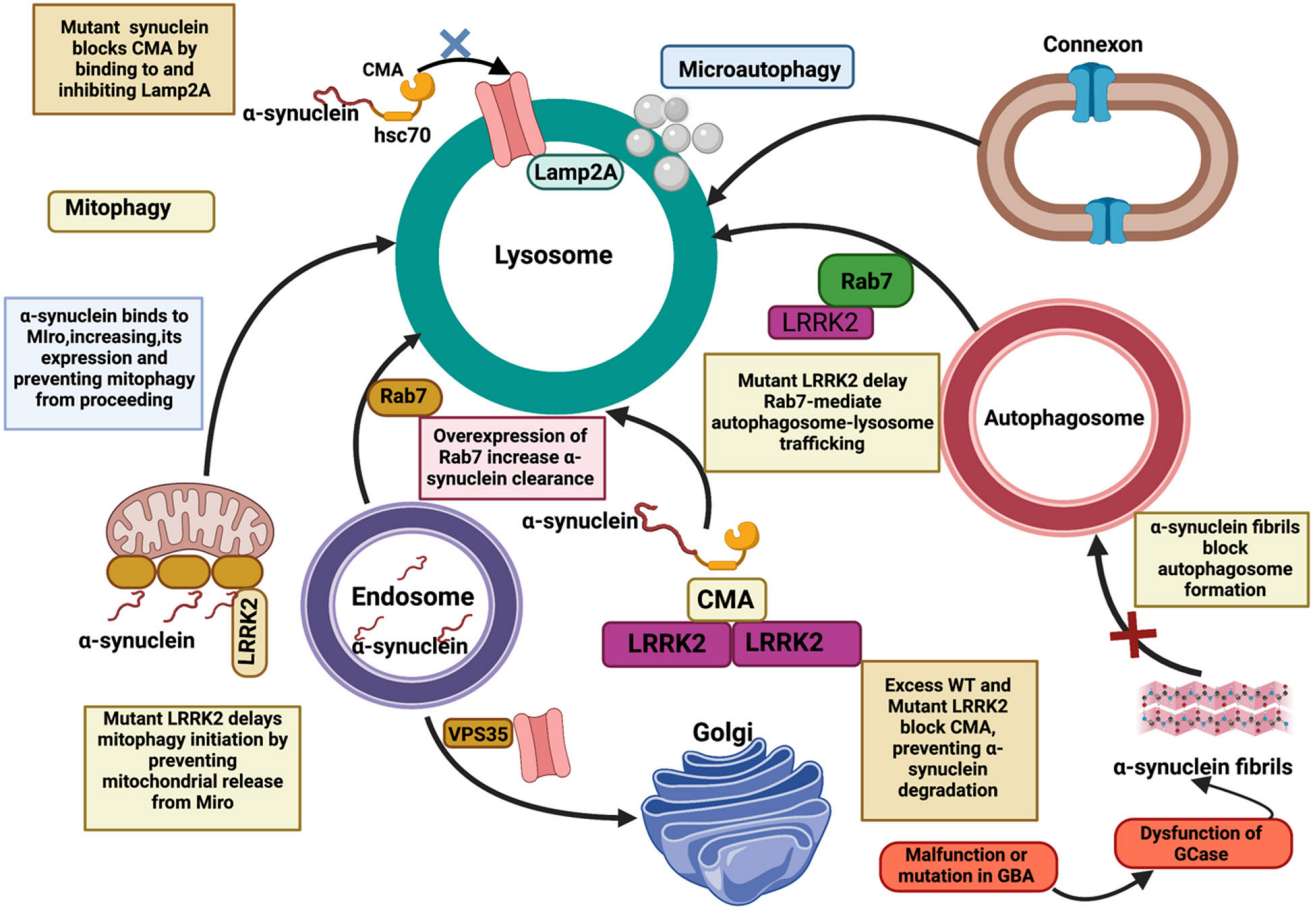
Cross connection between α-synuclein-GBA-LRRK2 axis and ion-channels/gap-junctions to develop PD. This is an example of convergent processes between LRRK2, GBA, and α-synuclein, with both impacting the autophagy-lysosomal system but acting on different targets within the system. CMA and autophagosome formation can be blocked by mutant or aggregated α-synuclein, and mutant LRRK2 can likewise impede CMA, impair mitophagy, and delay autophagosome trafficking. Malfunction in GBA can lead to the production of less active or inactive GCase thereby leading to aggregation of α-synuclein. This LRRK2-GBA-α-synuclein axis can also interfere with diverse neuronal ion-channels and gap-jucntions to PD pathogenesis. CMA Chaperone-mediated autophagy, Lamp2a lysosome-associated membrane protein type 2a, GCase β-glucocerebrosidase, GBA gene encoding GCase.

LRRK2 contains a Ras of complex (ROC) GTPase domain, *C*-terminal of ROC (COR) linker region, and serine/threonine kinase domain, *N*-terminal ankyrin domain, a leucine-rich repeat (LRR), and a *C*-terminal WD40 domain^128,129^. All these domains are responsible for diverse array of protein-protein interactions. As many as eight pathologic LRRK2 mutations (G2019S, R1441G/H/C, I2012T, Y1699C, I2020T, and N1437H) are linked with PD^94,130,131^. LRRK2 can get self-phosphorylated with the help of a P-loop with a DFG (conserved residues Asp–Phe–Gly)-APE motif, thereby regulating its functionality as a kinase^132,133^. α-synuclein directly interacts with LRRK2 thereby they both help to regulate each others’ functionalities (Fig. 10). α-synuclein can also aggregate thereby forming oligomers which then form fibrils to generate LB during physiological or other stress^134,135^. S129 of almost 90% of α-synuclein in LB gets phosphorylated in PD patients^136,137^. Since LRRK2 is a serine-threonine kinase, its hyperactive mutant G2019S LRRK2 can phosphorylate α-synuclein, thereby aggregating the latter and promoting apoptosis which is reversed by the inhibitors of LRRK2^138–142^. In contrast to this, there are reports that in vivo (at least in mice model), α-synuclein is not a substrate for LRRK2 kinase^143,144^. Moreover, a deletion-based double transgenic animal study has indicated that the LRRK2 kinase is not responsible for promoting A53T α-synuclein-driven neuropathophysiology^143^. At the same time, many other kinases like G-protein coupled receptor kinases, casein kinases, and polo-like kinases, are all responsible for phosphorylating α-synuclein^145–149^. LRRK2 and α-synuclein have been co-immunoprecipitated from transfected HEK293 cells under oxidative stress^141^, from brain tissue extracts of human PD and dementia with Lewy body (DLB) patients, but not from age-matched control brains^140,141^. All these effects were confirmed with the help of studies in transgenic mice, primary neuron-expressing mutant G2019S LRRK2^150,151^, and in human induced pluripotent stem cell (iPSC)-derived neurons from G2019S *LRRK2* carriers^151^. While these research reports conceptualized the effect of LRRK2 in α-synuclein-mediated cellular toxicity and a link between themselves, their exact mechanism of interaction is unclear to date. However, chaperones have been experimentally proven to be expected intermediates between LRRK2 and α-synuclein.

LRRK2 is also responsible for lysosomal protein trafficking and morphology. For example, PD patients’ fibroblasts carrying its related mutations resulted in dysregulated lysosomal morphology due to an enhanced influx of Ca^2+^ into the lysosome with the help of a diverse array of associated ion-channels^152^ (Fig. 10). Influx of this calcium can be reduced with the help of inhibitors of the two-pore calcium channels that are expressed over all acidic vesicles including endosomes and lysosomes of the E-L system^153,154^. Knocking out of LRRK2 in mice model resulted in deregulated clearance of proteins and over-accumulation of α-synuclein, thereby increasing the apoptotic cell death and oxidative impairments^155^. Overexpressed G2019S LRRK2 could reduce degradation capacity and enlarged lysosomes in astrocytes due to enhanced kinase activity of LRRK2^156^. Similar was the case in a study involving primary neurons cultured from G2019S LRRK2 knock-in mice which was diminished by inhibitors of LRRK2^156^. As many as fourteen Rab family members have been discovered to be phosphorylated by LRRK2 in their switch-II domains which is proof of a link between LRRK2 and the E-L system concerning its involvement in PD^157^. Rab3a and Rab8a can aid vesicular trafficking from the ER to the Golgi apparatus, thereby reducing α-synuclein-dependent cytotoxicity in animal models of PD^158^. Rab7 on the other hand is involved in the maturation of the autophagosome and early-to-late endosomes^158,159^. In an experiment in *Drosophila* Rab7 was found to connect LRRK2 and α-synuclein, as the former enhanced the clearance of α-synuclein aggregates, decreases cellular death, and protected locomotor malfunctions^160^. Nuclear factor erythroid-2 related factor (Nrf2), being an antioxidant protein, can be a promising drug target for neuronal damages^161^. Activation of Nrf2 reduces toxicity associated with LRRK2 and α-synuclein by enhancing the accumulation of LRRK2 in inclusion bodies and subsequently decreases its activity in other parts of the neuron^162^. It emphasizes that mutant LRRK2 may function as a negative regulator of autophagy thereby leading to α-synuclein deposition in DA neurons. α-synuclein can dysregulate E-L system thereby accelerating α-synuclein deposition using a feedback mechanism.

Gaucher disease occurs due to the loss of function of the lysosomal enzyme β-glucocerebrosidase (GCase), and the consequently increased level of glucosylceramide^163,164^. Such patients and carriers of heterozygous mutations in *GBA* (gene encoding GCase), are at higher threat to develop PD^165–168^. It is also now evident that accumulation of glycosphingolipids due to dysfunction of GCase can also enhance deposition of α-synuclein^169^. Such experiments were done in primary cultures of human iPSC^170^, and in rat brain^171^. Downregulation of GCase is further linked with reduced production of Beclin 1 (involved in autophagy). This was controlled via inactivating protein phosphatase 2A^171^. Combining all these findings, it can be postulated that a reduction in lysosomal enzymes can initiate a feedback mechanism by which inhibition of autophagy can be achieved thereby inhibiting upstream lysosomal functions. There is a controversy between the increase and decrease in GCase activity due to LRRK2 mutations, respectively^172,173^. It indicates that G2019S LRRK2 mutations are responsible for different pathological pathways. Hyperactivation of GCase triggers removal of α-synuclein and reversal of lysosomal activities in iPSC-derived human midbrain DA neurons from patients with *GBA*-related PD or idiopathic PD^174^. Downregulation of LRRK2 enzyme activity was seen to hyperactivate GCase in DA neurons obtained from patients with either *LRRK2* or *GBA* mutations, thereby decreasing the piling up of α-synuclein in such nerves^172^. Hyperactivation of LRRK2 can also result in Y307 phosphorylation and inactivation of Rab10, which in turn leads to a reduction in GCase activity. The latter can be prevented with inhibitors of LRRK2. Results showing that α-synuclein can hinder the lysosomal activity of GCase in neurons and brain tissue of idiopathic PD patients^170^ in turn convinced that the lysosome is a convergent organelle where dysfunctional LRRK2 and/or α-synuclein have destructive effects on lysosome function. The latter results in neurodegeneration like PD. Dysregualtion of this LRRK2-α-synuclein-GBA axis can also justify the increased tendency of DA neurons, since VPS35 and LRRK2 are crucial for vesicular trafficking and deregulation in these proteins may result into mistargeting of the dopamine transporter, DAT, thereby abnormal dopamine metabolism and oxidation (Fig. 10)^175^. The later in turn is reported to downregulate GCase^176^ and thus indicating the possibility of a negative feedback loop, making DA neurons uniquely vulnerable to degeneration.

### How do α-synuclein/GBA/LRRK2 interfere with the function of ion-channels/gap-junctions thereby leading to PD pathogenesis?

LRRK2 controls Ca^2+^ influx via controlling the voltage-gated Ca^2+^ (Ca_V_) channels. Presynaptic Ca_V_2.1 channels in turn stimulate neurotransmitter release. Like LRRK2, many other protein kinases, G-proteins, and Ca^2+^ binding proteins are also key regulators of Ca_V_2.1. Recently, Cade Bedford et al.^177^ discovered that LRRK2 protein interacts with specific synaptic proteins and influences synapsis. As synaptic proteins interact functionally with Ca_V_2.1 channels, synaptic transmission was seen to be triggered by Ca^2+^ entry via Ca_V_2.1. Ca_V_2.1 channel properties were measured using a whole-cell patch-clamp in HEK293 cells transfected with Ca_V_2.1 subunits and various LRRK2 constructs. Cade Bedford et al.^177^ results demonstrated that both WT and G2019S LRRK2 mutants result in a significant enhancement in whole-cell Ca^2+^ current in comparison to cells expressing only Ca_V_2.1 channel. Moreover, LRRK2 expression resulted in a significant hyperpolarizing shift in voltage-dependent activation while having no significant effect on inactivation properties. These Ca_V_2.1 functional alternations are probably due to LRRK2 since co-immuno-precipitation of LRRK2 and the β3 Ca_V_ channel was observed by scientists^177^. Moreover, Ca_V_2.1 channel activities are dependent on LRRK2 as evident from reversing effect by use of LRRK2 inhibitors. Interestingly, LRRK2 also augmented endogenous voltage-gated Ca^2+^ channel function in PC12 cells suggesting other Ca_V_ channels could also be regulated by LRRK2. Overall, these findings support a novel physiological role for LRRK2 in regulating Ca_V_2.1 function that could have implications for how mutations in LRRK2 contribute to PD pathophysiology. Moreover, overexpression of Kv2.1 channel can result in the reduction of DA in the striatum terminals of DA neurons and SN^84^. They can reduce K^+^ level thereby stimulating enzymes like nucleases and proteases intracellularly, leading to cell death^85^. This in turn also increases the intracellular Ca^2+^ concentration thereby increasing the Ca^2+^ influx into the cell, leading to cell damage and thereby linking with the flow of K^+^ inside the cell, hence causing PD^86^. Also, constitutive binding of calmodulin to the closer proximity of the C-terminus detects the action of Ca^2+^. LRKK2 mediated phosphorylated calmodulin influences susceptibility of SK channel. Calmodulin after binding to Ca^2+^, activates SK channels leading to a change in the membrane potential of a cell, thereby reducing its excitation^105^(Fig. 8). KATP channels also increase the Ca^2+^ concentration in nerves connected to NMDA receptor molecules^117^, thereby increasing the ROS generation which influences neuronal stimulatory action, increasing the amount of α-synuclein thereby further damaging thousands of neurons and causing induction of PD^118^. α-synuclein, is a type of KATP channel with having presynaptic neurons, functions informing and maintaining synapses^178,179^. α-synuclein present outside the cell membrane may be transferred from injuries to normal nerve cells by the prion-like mechanistic pathway, and this cellular transduction was observed in PD affected brain. In the striatum of mice, the KATP channel was activated by γ-aminobutyric nerve cells to reduce γ-aminobutyric acid concentration, further decreasing GABA receptor transmission on glutamate/aspartate neurons. Therefore, the Ca^2+^ level increases and triggers the α-synuclein secretory mechanism. Increased level of SUR1-mRNA in α-synuclein transfected MES23.5 cells and selectively activating Kir6.2/SUR1 in the SN leads to degenerated DA nerve cells; which proves that SUR1 plays a promising role in PD^180^. KATP/α-synuclein channel is expressed in the islet-β cell for controlling the secretion of insulin, thus interacting with Kir6.2 in ISGs to reduce the level of insulin. Misfolding in α-synuclein causes T2DM and PD by spreading prion-like activity and apoptosis^181^. Kir6.2-antisense oligo-DNAs were administered into GP, for the case of 6−OHDA hemiparkinsonian rat, which reduced the expressibility of Kir6.2-mRNA, thereby reducing apomorphine-induced the other side turns in the models. Pre-treatment of rodents with 6−OHDA induced abnormalities in PD with glibenclamide shows improvements in severe behavioral-symptoms^182^. Use of NaHS reduced 6−OHDA related behavioral symptoms and cellular damage of DA cells in the SN^183^. According to the clinical trials database, disease-modifying drugs namely antioxidants, botanicals, cell replacement therapies, mitochondrial function enhancers, GBA modifiers, glucagon-like peptide-1 agonists, immunotherapy, kinase inhibitors, neurotrophic factors^184^, are under clinical trials for the treatment of PD.

Nevertheless, LRRK2, α-synuclein, and GBA, all target the gap-junctions and ion-channels of the CNS to produce PD pathogenesis which of course needs further attention to understand the underlying mechanism. This might be an innovative way of therapeutic strategy to treat PD patients.

## Therapeutic interventions for PD involving ion-channels and gap-junctions

**Fig. 11:**
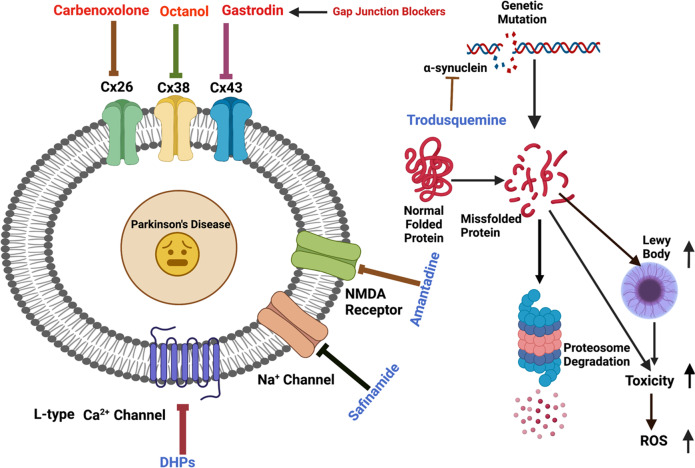
Pictorial depiction of different blockers of ion-channels and gap-jucntions to treat PD. While DHPs and Safinamide block Ca^2+^ and Na^+^ channels, respectively, Amantadine blocks NMDA receptor to treat PD. Similarly, Carbenoxolone (CBX), Octanol, and Gastrodin can block Cx26, Cx38, and Cx43, respectively as a treatment for PD. Trodusquemine can prevent aggregation of α-synuclein and related mutations to prevent Parkinsonism. DHPS Dihydropyridines (DHPs), ROS Reactive Oxygen Species.

To explore PD treatments, it is indeed necessary to understand the varied gradients of advancement in PD sufferers, which indicate the medical (and pathologic) diversity of the illness. A growing knowledge of the etiology and pathogenesis of PD has evolved the theories regarding novel neuroprotective treatments which can be effectively implemented in the prodromal stage. Various drugs that are employed in the treatment of PD are tabulated in Table 2 and shown pictorially in Figs. 9 and 11. Additionally, cell-replacement therapies were found to be advantageous over fetal cell-derived therapies^185^, in developmental and stem cell biology that comprises DA neurons derived from human pluripotent stem cells. However, improper knowledge about the treatment of PD, choice of inaccurate animal models and unvalidated biomarkers, and limitations in trial design restrict the development of effective neuroprotective therapies for PD. Novel transgenic animal models, adaptive and delayed-start trial designs, and identification of potential serum, cerebrospinal fluid, and neuroimaging biomarkers facilitates the development and testing of effective disease-modifying therapies (Table 3).

**Table 2:** Drugs that are involved in the treatment of PD.

Drugs	Mode of action	Side effects	Adverse effects	References
L-DOPA	DA protagonist	Increases DA levels	Short of breath, vomit, lower BP, tired, drowsy.	^214–216^
Selegiline	MAOB inhibitors	Maintaining Levo-DOPA	Dizzy, dried mouth, insomnia, muscular pains, rashes, shortness of breath, constipation, headaches, tachycardia, arrhythmia, hallucination	^217^
Creatine	Boosting mitochondria	Antioxidants, prevents MPTP induced apoptosis	Short of breath, stomachpain, diarrhea, muscular cramp, swell in specific body parts, weight gain.	^218^
Bromocriptine, Apomorphine	DA protagonist	Increase DA concentrations	Drowsy, short of breath, vomit, dried mouth, dizzy, swelling in limbs, fainting, decrease in BP, confusions, hallucination	^219^
Pramipexole, Ropinirole	Non-ergoline dopamine agonist, specific to D2 receptors	Peripheral and central dopaminergic stimulation	Dizziness, headache, nausea, and somnolence	^220^
Entacapone and tolcapone	Prevents DA degradation	Extends Levo-DOPA effect	Short of breath, diarrhea, orthostatic hypotension, urinaryproblems, dizziness, abnormal functioning of mitochondria	^221^
Amantadine	Activates DAsynthesisation	Increase DA concentrations	Confusions, urinary problems, feeling dizzy, fainting, problems in the sense organs, swelling in the limbs	^222^
Rofecoxib	COX2 inhibitor	Prevent inflammation	Spinal pain, diarrhea, dizziness, migraines, lack of energy, running nose, swelling in limbs, blur in eyesight, intestinal problems.	^223^
Pimavanserin (ACP-103)	Blockage of Serotonin receptor	Decreases L-DOPA related problems	Hyperprolactinemia, improper functioning of menstrual and sex organs, akathisia, distressful motor disturbance, tiring body	^224^
Quetiapine	Blockage of 5-HT2 and D2 receptor	Reduces agitated psychotic symptoms	Agitation, dizziness, tremors, anxiousness, hypertonia, indigestion, involuntary movements, worries, hyperkinesia, increased-libido, abnormality in gait, myoclonus, apathy, ataxia, hemiplegia, aphasia, BS (buccoglossal syndrome)	^225^
CoQ-10 (Ubiquinone)	Improved functioning of mitochondria	Antioxidants lower disease progression in the earlystages	Low BP, hemorrhage, skin itchiness, shortness of breath, vomit, headaches, migraine, shortage of breath, spinal and chest pain, constipation, coughs, diarrhea, dizziness, faints, falls, fatigues, hearing problems, mild stroke, indigestion, insomnia, muscularpain, night-sweat, pharyngeal infection	^226,227^
Entacapone, tolcapone	COMT inhibitor	Inhibit dopamine degradation	Diarrhea, shortness of breath, sleep disturbances, dizziness, urination disorders, pain in the abdomen, lower BP, hallucination	^228^
S-Adenosylmethionine (SAM)	Phospholipid-methylation and increases neural-communication	Improve DA transmissibility, decrease depression	GI diseases, GERD, and anxiousness	^229^
Carbenoxolone	Inhibitors of Gap junctions Cx38, Cx26, Cx43, Panx1 particularly	Disruption in electrolyte balance such as sodium retention and hypokalemia	Cardiac arrest left ventricle failure, visual impairments	^49,230^
Tonabersat	Inhibts connexin hemichannels	Discomfort and drowsiness	Severe adverse effects appeared infrequent, as well as none individual dropped out of any trial due to them	^231^
ketamine, propofol and dexmedetomidine	In vivo, tight junctions interaction with astrocytes is considerably blocked.	Dissociation elevated blood pressure	None of the drugs include adverse side effects	^188,232^
Gastrodin	It current plays an inhibitory action with the expression of Cx43 and also to some extent the activity of hemichannels	Drugs is having very less side effects.	The drug includes no adverse side effects.	^233^
Cannabinoids (CB’s)	In neuro-inflammatory circumstances, CBs are critical chemicals to prevent excessive activation of connexin hemichannels and pannexin channels.	Obesity, elevated level of calorie intake	Impaired cognition	^234^
Apamin	Inhibits SK channels	Not available	Not available	^235^
Isradipine	Targets LTCCs	Increased dosages might exacerbate trembling, induce disorientation, even induce vomiting, alterations in blood, tension	Same as the side effects mentioned	^236^
Safinamide	Inhibits Ca^2+^ channel activity	Elevated increase of high blood pressure	Dyskinesia, Dyspepsia, Orthostatic hypotension	^235^
4-Aminopyridine	Blocks Kv channels	Not available	Not available	^235^

**Table 3:** Latest technologies used for detecting and neuroimaging PD.

Technologies	Applications in PD	References
*Cueing Devices*	PD patients receives signals to modify their motions in right directions. Patients receive sensory feedback from vibrating sensors and/or electric induder.	^237^
*Biopotential Devices*	A biopotential device is integrated into a wearable device like EEG, ECG, and magnetoencephalogram for the treatment and monitoring of PD patients.	^237^
*Optical Motion Tracker*	This helps to monitor the movements of the POD patients with the help of a radio frequency and/or optical device.	^237^
*Audio Recording*	PD patients’ speech and swallowing like indications are recorded using a mobile phone or a voice recorder for diagnosis and prognosis purposes.	^237^
*Video Recording*	Recors gait and motor issues in PD patients.	^237^
*Force/Pressure*	Force/pressure sensing devices can be inserted into gait mats to monitor the gait quality of a PD patient.	^237^
*Smart phone*	A smart phone screen can record handwriting, its microphone can record speech, and the camera can record PD patient’s motions.	^237^
*X-ray computed tomography and brain MRI*	It can diagnose structural lesions present with vascular pathophysiology/neoplasms and detects the amount and location of brain-atrophy.	^238^
*PET or SPECT*	It can provide images for DA related activities in the presynaptic neurons to diagnose and monitor PD.	^238^

### Mechanism of action of blockers of gap-junctions to treat PD

A wide range of substances has been documented to work as gap-junction blockers (GJBs). Nevertheless, those chemicals are often rarely specific and can also potentially adhere to a wide range of different sites, resulting in multiple phenotypic expression side-effects. A variety of GJBs was developed for use in PD^49^. GJBs such as carbenoxolone (CBX) and octanol strongly suppress the activation of Cx38- and Cx26-based tight junctions. CBX was also demonstrated to inhibit α-synuclein aggregation inside a rotenone-induced PD mouse **(**Fig. 11**)**^186^. This result is analogous to the suppression of inflammatory response and oxidative damage caused by α-synuclein accumulation, which is attributable in addition to inhibitory actions of CBX on Cx43 tight-junctions^187^. Tonabersat is a cis-benzopyran chemical that blocks connexin hemichannels. Tonabersat has been shown to lower Cx26 activity inside the cortex by blocking the p38-mitogen triggered protein kinase pathway. Additionally, it can effectively attach to and inhibit Cx43 hemichannels^49^. In vitro, anesthetics such as ketamine, propofol, and dexmedetomidine dramatically inhibit tight-junctions interaction amongst astrocytes^188^. Gastrodin, a Chinese medicinal herb component, was also utilized as a GJB. In rotenone-induced PD rodent models, gastrodin inhibits Cx43 synthesis, activation, and cellular interaction. Interestingly, gastrodin inhibits the symptomatic characteristics of PD inside this animal paradigm^189^. The lowered expression of Cx43 by gastrodin may affect Cx43 hemichannel activity, which might explain the desirable outcomes^190^. Cannabinoids (CBs) have been considered as possible treatments for PD. CBS also has beneficial effects on neural tissues through altering gap-junctional signaling. Anandamide, for example, which is an endogenous arachidonic acid derivate, can activate neuronal CB receptors by suppressing gap-junctional conductivity throughout astrocytes^49^. According to the clinical trials database, disease-modifying drugs namely antioxidants, botanicals, cell replacement therapies, mitochondrial function enhancers, GBA modifiers, glucagon-like peptide-1 agonists, immunotherapy, kinase inhibitors, neurotrophic factors^184^, are under clinical trials. Dizocilpine, 4-AP, has shown promising results in the pre-clinical studies and needs further investigation. Drugs such as Safinamide^191^, Zonisamide^192^, Amantadine, etc. have opened up the scope for further exploration of ion-channels for slowing down the disease progression^193^.

### Mechanism of actions of blockers of ion-channels to cause PD

However, the difficulty with ion-channels as a target is due to their wide expression in the brain, hence activation or inhibition of the ion-channels might lead to many certain side effects if not specific. Therefore, drugs and small molecules need to be developed in a very selective manner. This is where comes the necessity of crossing the blood-brain barrier (BBB). Although direct injection of drugs in the required brain regions, was found to be extremely invasive and expensive, it was found to be a promising alternative. In this regard, assisted transport of drugs through BBB was performed using specialized nanoparticles or receptors via endocytosis or transcytosis^194^. Several studies have shown the effectiveness of using nanoparticles for delivering drugs specifically to dopaminergic neurons in the animal models of PD. In use of lipid nanoparticles covered with lactoferrin to deliver GDNF in 6−OHDA lesioned rats^195^, were shown to improve the motor phenotype and TH immunostaining^195^. In the same manner, shRNA against the SNCA gene has been delivered to MPTP-treated mice using Fe_3_O_4_ nanoparticles. These magnetic nanoparticles used NGF to bind the tyrosine kinase receptors expressed specifically on neurons for binding and prevented MPTP-mediated neurodegeneration^196,197^. A very recent study reported the enhanced expression of CACNA1D, a gene encoding a subunit of the voltage-gated calcium channels, in PD microarray data^197^. Therefore, there is a ray of hope that shortly ion-channel subunits might serve as useful biomarkers and targets for the treatment of PD as shown in Fig. 11.

Apart from the two major modes of action of the ion-channels, i.e., (1) pore plugging, and (2) allosteric binding, there are other mechanisms by which inhibition of ion-channels occurs. In the pore plugging mode of action, there is a physical obstruction in the transport of ions through the channel pore via the binding of inhibitors in the pore region. However, this method of inhibition is a reversible process, and the normal functionality of this channel can be restored after washing out the inhibitor. Non-allosteric binding of STX and TTX plug the NaV channel from the extracellular part of the pore. At times when the inhibitor passes first through the cell membrane, plugging occurs from the intracellular side of the channel. TeA (Tenuazonic acid) and QA (Quaternary ammonium) ions inhibit the KV channels from the intracellular side, by open channel inhibition, which can take place only when the channel is in an open state and not when closed. Depolarization of the cell membrane opens up the voltage gate for TeA to enter the central cavity and become lodged to plug the channel.

On the other hand, the allosteric mode of binding requires a specific binding site on the extracellular part of the pore where an allosteric inhibitor binds to the channel, thereby causing conformational changes, hence preventing the normal functionality of the channel. This mechanism can be relatable to the way agonist and antagonist bind to the receptors of the outer membrane, and similar to the way nAChR undergoes ligand-binding. The allosteric inhibitor binds to and blocks the channel in a nonconducting conformational state. Dihydropyridines (DHPs), L-type Ca^2+^ channel (CaV1) inhibitors, and most natural toxins use this method of channel inhibition. In the case of voltage-gated channels, it is often located somewhere on the voltage-sensor domain (S4) or the inactivation ball (Fig. 11). The voltage-sensor domain is partially exposed to the extracellular space, which makes the binding easier. Such modulators interfere with the voltage-sensor and thus modulate the ion-channel gating. For this reason, they are often referred to as voltage-sensor modulators or gating modifiers. Gating modifiers have a favorite binding site for inhibitors and channel modulators located on the voltage-sensor domain (S4), thereby leading to a channel activation-inhibition mechanism, or even an increased, delayed, or complete inactivation process.

To summarize, ion-channels play a vital role in driving PD pathology. There is numerous emerging evidence that proves the usefulness of ion-channels as a therapeutic target for PD. However, to effectively utilize ion-channel modulation for therapies, it is important to deeply understand the alteration of ionic homeostasis with the disease course. Simultaneously, there is a need to understand the interlinked pathways which regulate the expression and functioning of the various channels that will pave the way for the development of therapeutics to bring about the much-needed relief to PD patients.

## Conclusion and future perspectives

PD has afflicted people over many generations and is a debilitating neurodegenerative disorder. To date, much of the research has been focused on unraveling the molecular details of α-synuclein and tau proteins. It is an urgent need to look into various systemic aspects of the disease. In this current review article, we have shed light on new areas of research within PD. The importance of neural cell-junctions and ion-channels may open up new avenues to better diagnose and treat PD. Through a thorough literature survey, we put forward several burning questions for the better management of this disease. Mimicking mammalian Parkinsonism is an important aspect of this and certain techniques and methodologies need to be developed^38^, especially for proper understanding of this complicated ailment. Some of these are: (i) Developing different models of mammals which could be a combination of many modular organisms for genetic and toxin effects; (ii) The use of non-dopamine-drugs, namely α2-adrenergic protagonists, adenosine-A2a protagonists, is needed for PD diagnosis; (iii) Usage of IPX-066, XP-21279, and Opicapone, MAO inhibitors such as safinamide to reduce depression and dyskinesia; (iv) Targets ALP-technique and UPS-technique in PD treatments; (v) Developing transplantation therapy using dopamine nerves from ISCs (Interval Specific Congenic Strains); (vi) miRNA/siRNA based approaches for the inhibition of mRNA of abnormal folding in protein complexes; (vii) Using CRISP-Cas9 technique in correcting mutant-genes^198^; (viii) To understand if PD shows a dual disorder in animals and humans; (ix) Using electrophysiology experiments to further understand PD; (x) Development of different ion-channels to deal with neuroinflammation during Parkinsonism, etc.

Nevertheless, several issues need to be addressed before effective gene therapy can be safely used to treat PD. Major efforts are needed in identifying genetic materials for rectifying, developing safe-maker genes, and modulation of compact expressibility of genes in the central nervous system^199^. Increased barrier-permeability in gut-lumen allows reduction of negatively regulated brain neurons and neurons of the GI tract, thus providing a diagnostic pathway in Parkinsonism and GI-related diseases^198^. Novel exosome-based diagnostic methodology from patients’ biofluids has shown the potential to diagnose various neurodegenerative diseases including Parkinson’s^200,201^ and Alzheimer’s disease^201^. An in vivo TST (triple sugar test), fecal-biomarkers, ex vivo PT, and tissue-staining techniques allowed for comparing and validating different techniques for pathological methods in the treatment of PD. The reductionist-model system can help elucidate the pattern of processes and pros and cons of these methodologies, thus identifying microorganisms and MF (Microbial Factor), which could have promoted α-synuclein pathways in nerve cells. Patients with fibroblast-derived iPSC are another approach for future studies. iPS cells were divided into three-dimensional intestinal organoids, also known as mini-guts^202^. A three-dimensional epithelial culture may be essential for EPA (epithelial permeability analysis)^202^. iPSC allows cultures of intestinal organoids with ENS at the bottom of cells that were collected for controlled experiments. iPSC-derived intestinal organoids lacked epigenetics and displayed immaturities^202^ with lacking related aspects of Parkinsonism. From a different aspect, it can be said that the mini-gut models were utilized for comparing with the genes of the patient, which could have been one of the parameters contributing toward the integration of mucosal barriers, which is yet to be studied under PD.
